# Identification of Key Tissue-Specific, Biological Processes by Integrating Enhancer Information in Maize Gene Regulatory Networks

**DOI:** 10.3389/fgene.2020.606285

**Published:** 2021-01-11

**Authors:** Maud Fagny, Marieke Lydia Kuijjer, Maike Stam, Johann Joets, Olivier Turc, Julien Rozière, Stéphanie Pateyron, Anthony Venon, Clémentine Vitte

**Affiliations:** ^1^Université Paris-Saclay, INRAE, CNRS, AgroParisTech, GQE – Le Moulon, Gif-sur-Yvette, France; ^2^Centre for Molecular Medicine Norway (NCMM), Nordic EMBL Partnership, University of Oslo, Oslo, Norway; ^3^Department of Pathology, Leiden University Medical Center, Leiden, Netherlands; ^4^Plant Development and (Epi) Genetics, Swammerdam Institute for Life Sciences, University of Amsterdam, Amsterdam, Netherlands; ^5^LEPSE, Univ Montpellier, INRAE, Institut Agro, Montpellier, France; ^6^Université Paris-Saclay, CNRS, INRAE, Univ Evry, Institute of Plant Sciences Paris-Saclay (IPS2), Orsay, France; ^7^Université de Paris, CNRS, INRAE, Institute of Plant Sciences Paris-Saclay (IPS2), Orsay, France

**Keywords:** enhancers, gene regulatory networks, *Zea mays*, transcription factor binding sites, transposable elements, TIR transposon, mite, husk

## Abstract

Enhancers are key players in the spatio-temporal coordination of gene expression during numerous crucial processes, including tissue differentiation across development. Characterizing the transcription factors (TFs) and genes they connect, and the molecular functions underpinned is important to better characterize developmental processes. In plants, the recent molecular characterization of enhancers revealed their capacity to activate the expression of several target genes. Nevertheless, identifying these target genes at a genome-wide level is challenging, particularly for large-genome species, where enhancers and target genes can be hundreds of kilobases away. Therefore, the contribution of enhancers to plant regulatory networks remains poorly understood. Here, we investigate the enhancer-driven regulatory network of two maize tissues at different stages: leaves at seedling stage (V2-IST) and husks (bracts) at flowering. Using systems biology, we integrate genomic, epigenomic, and transcriptomic data to model the regulatory relationships between TFs and their potential target genes, and identify regulatory modules specific to husk and V2-IST. We show that leaves at the V2-IST stage are characterized by the response to hormones and macromolecules biogenesis and assembly, which are regulated by the BBR/BPC and AP2/ERF TF families, respectively. In contrast, husks are characterized by cell wall modification and response to abiotic stresses, which are, respectively, orchestrated by the C2C2/DOF and AP2/EREB families. Analysis of the corresponding enhancer sequences reveals that two different transposable element families (TIR transposon *Mutator* and MITE *Pif/Harbinger*) have shaped part of the regulatory network in each tissue, and that MITEs have provided potential new TF binding sites involved in husk tissue-specificity.

## 1. Introduction

Enhancers are key regulators of the spatio-temporal expression of genes in eukaryotes, in particular during development (Spitz and Furlong, 2012; Weber et al., 2016). Their regulatory effect is mediated by the binding of transcription factors (TFs), which interact with target gene promoters through 3D-loops over distances reaching several dozens of megabases in some species (Ricci et al., 2019; Robson et al., 2019). The binding of a single TF is often not sufficient to activate the expression of a gene, and generally several TFs act together to increase or decrease the regulatory potential of a given enhancer (Spitz and Furlong, 2012). Groups of enhancers characterized by similar content in transcription factor binding sites (TFBSs) have been shown to co-regulate genes involved in the same biological pathways, thus shaping a complex regulatory network controlling the tissue-specific expression of genes involved in particular biological functions (Vermunt et al., 2014; Chen et al., 2018). While enhancers have been identified as key players in the wiring of the developmental gene regulatory network in mammals (Vermunt et al., 2014; Cvekl and Zhang, 2017), this question remains largely unexplored in plants (Weber et al., 2016).

Recent combined analyses of DNA methylation, chromatin accessibility and histone marks have led to the genome-wide characterization of thousands of putative active enhancers in plants (Zhu et al., 2015; Oka et al., 2017; Wang et al., 2017; Zhao et al., 2018; Li et al., 2019b; Lu et al., 2019; Ricci et al., 2019). Distance between each enhancer and its nearest gene varies strongly depending on the species, ranging from about 2 kb in *Arabidopsis thaliana* to 1 Mb in barley, and is largely correlated to genome size (Lu et al., 2019). In maize (*Zea mays* ssp. mays), 3D chromatin folding analyses showed that about 25%-40% of enhancers are not targeting their closest gene, and that 34% of enhancers potentially regulate several genes (Li et al., 2019a; Ricci et al., 2019). These results highlight the difficulty to identify the regulatory relationships between enhancers and their target genes in plants with large genomes.

How enhancers arise and rewire the gene regulatory network in plants is unclear. Transposable Elements (TEs) of various superfamilies have been proposed as a source of new regulatory elements (Percharde et al., 2020) and have been shown to be involved in the rewiring of gene regulatory networks for some key tissue-specific biological functions in animals (Chuong et al., 2017). In plants, examples of enhancers derived from a particular TE have been described (Studer et al., 2017; Barco et al., 2019; Shi et al., 2019), and a more general contribution of TEs to *cis*-regulatory elements has been highlighted in some species such as *Capsella grandiflora* (Uzunović et al., 2019) and maize, where at least a quarter of the thousands of putative enhancers were found to overlap TE annotations (Oka et al., 2017; Zhao et al., 2018). TEs influencing the response of nearby genes to abiotic stresses have also been described, for example in maize seedlings (Makarevitch et al., 2015), hinting for an important role of TEs in regulating the expression of genes involved in specific biological functions in plants. Nevertheless, whether TEs contribute to the emergence of tissue-specific gene regulatory networks in plants remains to be fully elucidated.

As of today, it remains both time-consuming and expensive to test the enhancer-target gene regulatory relationship at the genome-wide level using molecular biology approaches such as enhancer reporter assays and CRISPR-Cas manipulation. By offering approaches to model *in silico* the regulatory relationships between heterogeneous components such as TFs and genes, systems biology provides a powerful and cost-effective alternative. Classical co-expression networks allow to group TFs with potential non-TF target genes. However, they do not provide information about whether these regulatory relationships are actually possible in terms of binding of the TF to regulatory elements associated with the target genes. Integrating information about the genes *cis*-regulatory sequences, in particular about which TFBS they harbor, allows to connect TFs more directly to their potential target genes. This information can then be integrated with gene co-expression information to generate bipartite TFs-genes networks. Such systems biology approaches have contributed to decipher the role of promoter-binding TFs in the regulation of their target genes in tissues or cell cycle stages in fungi and animals (Glass et al., 2013; Lopes-Ramos et al., 2017; Sonawane et al., 2017; Kuijjer et al., 2019), and to identify the impact of disease on the wiring of tissue-specific regulatory networks in humans (Padi and Quackenbush, 2018). With recent advances in active enhancer characterization, TFBS annotation, and the generation of expression data from a large number of tissues, these systems biology approaches can now be used in plants and open new opportunities to study the regulatory role of enhancers during plant development. Improvement of genome sequences and TE annotation also allows for characterizing the part of enhancers driven by TEs, and therefore to investigate the potential role of TE sequences in rewiring gene regulatory networks in plants.

In this study, we investigate the interconnection between TFs, enhancers and target genes in maize tissue-specific gene regulation, by comparing the regulatory networks of two types of maize leaves at different developmental stages: immature leaves at V2 seedling stage and husks (bracts) at flowering. Taking advantage of enhancers that were previously predicted by Oka and colleagues to be active in these two organs (Oka et al., 2017), we analyze their TFBS composition. Using bipartite networks, we then integrate this information with relative genomic position of genes and enhancers together with transcriptomic data, to reconstruct tissue-specific TFs-genes regulatory networks. We identify key TFs that co-regulate groups of genes involved in biological functions crucial for tissue identity, and link these genes to the enhancers that regulate their expression. By analyzing sequences of these enhancers, we show that TIR transposon *Mutator* and MITE *Pif/Harbinger* families are involved in the tissue-specific expression in immature seedling leaf and husk, respectively. We also discover that MITEs harbor conserved sequences that are likely maize-specific TFBS, thus highlighting that TEs are important players in shaping regulatory networks in this species. An online queryable version of the networks is available at https://maud-fagny.shinyapps.io/TF-gene_network_Maize/.

## 2. Results

### 2.1. Husk and V2-IST-Specific Enhancers Are Enriched in Binding Sites Targeted by Different TF Families

**Figure 1:**
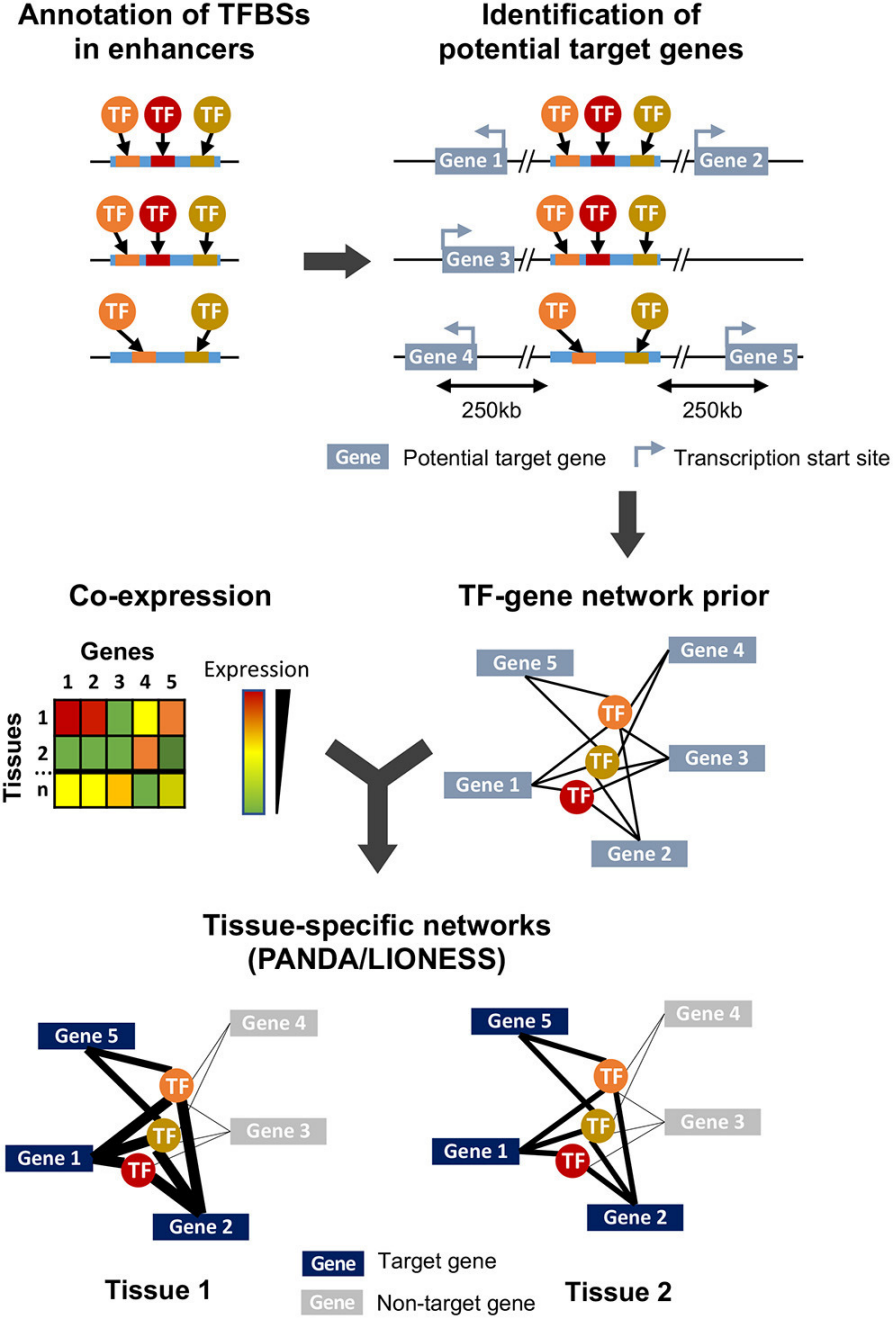
Overview of the study design and methodology. Enhancer sequences are first searched for TFBS motifs using FIMO. All genes whose transcription start site is within 250 kb of an enhancer are considered as potential targets, and combination of this genomic information and TFBS annotation leads to the generation of a TFs-genes prior network. In parallel, a gene-gene co-expression matrix is generated from expression data obtained from different samples (here, corresponding to different tissues) and integrated to the TFs-genes prior using PANDA and LIONESS to obtain tissue-specific regulatory networks.

We first aimed to characterize the TFBS content of active enhancers in husk and V2-IST (a description of these tissues is available in Supplementary Figure 1 and Supplementary Table 1). To this end, we extracted sequences of the 1,495 putative active enhancers (hereafter called “enhancers”) obtained from Oka and colleagues, among which 1,097 were found specifically active in husk, 175 specifically active in V2-IST, and 223 active in both tissues. We *in silico* annotated the TFBSs located in these enhancers by scanning for known plant TFBSs (Figure 1). After selecting for TFBSs with a Benjamini–Hochberg-corrected *p*-value below 0.01 (see section 4), we retained 18,348 TFBSs corresponding to 62 transcription factors. A table containing the annotation of TFBS in each enhancer is available in Supplementary Table 2. Among all enhancers, 489 (32.7%) did not harbor any significant TFBS, including 411 (37.5%) husk-specific enhancers, 32 (18.3%) V2-IST-specific enhancers and 46 (20.6%) shared enhancers. Using a resampling approach (see section 4), we tested for TFBS enrichment in enhancers of each tissue. We found that 60.6% of the V2-IST-specific enhancers, 60.6% of the husk-specific and 66.8% of the shared enhancers were significantly enriched for TFBSs compared to randomized sequences. On average, an enhancer contained 11.4 TFBSs (ranging from 0 to 255), which covered on average a total of 34.3 bp (ranging from 0 to 442 bp) or 2.5% of the enhancer sequence length. Because the JASPAR TFBS motif database used for this annotation is mostly based on ChIP-seq and DAP-seq data generated in *Arabidopsis thaliana*, we wanted to check whether the TFBSs we predicted in maize were supported by ChIP-seq and DAP-seq data in maize. The binding landscape of 32 TFs has been generated in maize leaves using DAP-seq (O'Malley et al., 2016; Galli et al., 2018; Ricci et al., 2019). Among our 52 TFs, only one had DAP-seq data for one of its maize ortholog: AT1G12630. All of the 18 TFBSs that we predicted for this TF co-localized with DAP-seq peaks according to the plant Epigenome browser (http://epigenome.genetics.uga.edu/PlantEpigenome/).

We then compared the TFBS content of enhancers active in husk and these active in V2-IST. Husk enhancers were enriched for binding sites corresponding to 15 TFs, mostly from the C2C2/DOF family (7), but also from the AP2/ERF (5), HD-ZIP (2), and bHLH (1) families. More precisely, in addition to the 3 TFs of the C2C2/DOF family that had significantly more binding sites in the husk enhancers, 4 were found only in husk enhancers (Benjamini–Hochberg adjusted *p*-value lower than 0.05, Supplementary Table 3). TFs from the C2C2/DOF family are known to be mainly involved in response to abiotic and biotic stresses, and are expressed in growing and mature leaves (Chen and Cao, 2015; Stelpflug et al., 2016; Hoopes et al., 2019). In contrast, V2-IST enhancers were enriched for binding sites recognized by TFs from the AP2/ERF family, with 17 TFs having significantly more frequent TFBSs in V2-IST enhancers than in husk enhancers (Benjamini–Hochberg adjusted *p*-value below 0.05, Supplementary Table 3). AP2/ERF TFs are known to be involved in plant development and growth, and transition to flowering (Gu et al., 2017), and are known to be expressed in seedlings (Stelpflug et al., 2016; Hoopes et al., 2019).

### 2.2. TFs-Genes Regulatory Interactions Recapitulate Tissue-Specific Enhancer Activity

To investigate the tissue-specific regulatory relationship between TFs and their target genes, we first built a prior network. In this prior, we considered that a gene was potentially targeted by a TF if an enhancer containing the TFBS recognized by this TF was located within 250 kb of the transcription start site of a gene (Figure 1, see section 4). This threshold was chosen based on the available data on maize intergenic open chromatin region-nearest gene distance distribution where 99% of open chromatin regions are located within 250 kb of a gene's transcription start site (Lu et al., 2019; Ricci et al., 2019). This is also in accordance with the distance observed in well-characterized enhancers and their target genes in maize, which can span up to 140 kb (summarized in Weber et al., 2016). All enhancers (i.e., these found in husk or in V2-IST and that contained at least one annotated TFBS) were used. Because about 25% of enhancers are indicated to be downstream of their target genes (Ricci et al., 2019), we included all genes independently of their genomic orientation. To build tissue-specific regulatory networks, we combined this prior with gene co-expression data (Glass et al., 2013; Kuijjer et al., 2019).

We had access to mRNA-seq data from husk and V2-IST (6 replicates each) from Oka et al. (2017). To obtain high confidence tissue-specific networks (Kuijjer et al., 2019), we enriched this dataset with mRNA-seq data that we generated from 11 tissues in triplicates (see section 4, Supplementary Figure 1 and Supplementary Table 1). In total, our merged dataset included 45 samples and a total of 46,430 genes. We retained genes expressed in at least 3 samples in at least 1 tissue (see section 4), corresponding to a total of 36,041 genes. Read counts were then normalized and corrected for the single-end/paired-end mRNA-seq data type (see section 4). As shown by correlation analyses (Supplementary Figure 2), replicates correlate more between themselves (average biological replicate Pearson correlation coefficient *R* = 0.95) than with any other samples (average inter-tissue Pearson correlation coefficient *R* = 0.83). Moreover, expression levels from the V2 inner immature tissues from both datasets (V2-IST and V2_L-I) are strongly correlated (average pairwise Pearson correlation coefficient *R* = 0.93). The principal component analysis of normalized RNA-seq data (PCA, Supplementary Figure 3) show that biological replicates cluster together, and that the data from both datasets are highly comparable. In particular, the V2-IST and V2_LF-I also cluster together in the PCA.

To build our network, we retained only enhancers carrying at least one TFBS corresponding to expressed TFs. Of the 62 TFs for which TFBSs were identified within the 1495 enhancers, 10 were not expressed in any of our samples and were therefore filtered out. In total, 971 enhancers carried at least one TFBS corresponding to one of the 52 expressed TFs. Out of the 36,041 genes, we identified a total of 8,054 potential target genes that were located within 250 kb of one of the 971 enhancers, and that we included in the prior. Among those, 6,459 (80%) were potentially targeted by a single enhancer, 1,269 (16%) by two enhancers and 326 (4%) were potentially targeted by three enhancers or more. Conversely, each of the enhancers had an average of 10 potential target genes (ranging from 1 to 42). Our prior gene regulatory network thus contained 52 TFs that had TFBS in one of the 971 enhancers, and 8,054 genes. Each gene was connected to an average of 8.6 TFs (ranging from 1 to 33), and each TF was linked to an average of 1,310 genes (ranging from 2 to 3,431).

We combined the prior gene regulatory network to the co-expression matrix obtained from the mRNA-seq normalized data from the 45 samples (13 tissues). We then built sample-specific gene regulatory networks using PANDA and LIONESS (Glass et al., 2013; Kuijjer et al., 2019), and obtained 45 sample-specific TFs-genes regulatory networks (Figure 1). These networks differ from co-expression networks. Here, a higher edge weight does not represent a higher expression correlation between a TF and its target gene. Rather, it captures a higher expression correlation of the genes targeted by the similar sets of TFs, and indicates a higher likelihood of a regulatory interaction between the TF and its target gene (Sonawane et al., 2017). We generated a 2D representation of the sample-specific networks using a uniform manifold approximation and projection for dimension reduction (UMAP) approach (Supplementary Figure 4), and compared it to the PCA results on gene expression data. As expected, samples from the same tissue cluster together on the UMAP, and the V2-specific regulatory networks generated from V2 growing leaves from the formerly and newly generated datasets cluster together. Notably, while in the PCA husk samples were isolated and located close to silk and internode tissues (Supplementary Figure 3), in the UMAP the husk-specific regulatory network was clustered with regulatory networks of other types of mature leaves, thus indicating stronger similarity in terms of gene regulatory network than gene expression levels between tissues that share similar developmental stages.

We then verified that known tissue-specific activation of text book examples of enhancers were retrieved by our network by examining the tissue-specific regulatory relationships between the TFs they were predicted to bind and their target genes. Among the three known enhancers included in the study of Oka and colleagues, two had known target genes mapped within 250 kb in the AGPv4 maize genome assembly: these of *tb1* and *bx1* (also known as DICE). The third one, *b1* enhancer, has not been assembled in AGPv4, and as such could not be included in our analysis. We found that the regulatory relationship between *tb1* and the TFs binding its enhancer is stronger in husk than in V2-IST (Supplementary Figure 5A). This is in accordance with former observations that the *tb1* enhancer is active in husk but not in V2-IST (Oka et al., 2017). In contrast, the regulatory relationship between *bx1* and the TFs binding its enhancer (DICE) were of similar strength in both husk and V2-IST (Supplementary Figure 5B), in accordance with the fact that DICE was shown to be active in both tissues. Hence, our approach, which uses a generic TFs-genes prior together with RNA-seq data from 13 tissues to build both husk and V2-IST networks, is able to retrieve the activated/unactivated states of these two known enhancers in each tissue specifically.

### 2.3. Tissue-Specific Regulatory Modules Highlight Different Biological Functions in Husk and V2-IST

Our first aim was to identify and biologically characterize regulatory networks that were differentially regulated between husk and V2-IST. To this end, we first performed a differential targeting analysis of the V2-IST and husk tissues by comparing the edge weights of the sample-specific networks between the two tissues (see section 4). We thus identified 2,075 genes that were more highly targeted by TFs in husk and 2,123 genes that were more highly targeted in V2-IST (Benjamini–Hochberg corrected *p*-value of 0.05). The 3,856 remaining genes were not significantly differentially targeted in any of the two tissues. Using a Gene Ontology enrichment analysis (Supplementary Table 4a), we found that genes with a higher TF-gene regulatory relationship in husk were enriched for biological processes related to post-transcriptional protein modification (GO:0006468, GO:0006486), regulation of transcription (GO:0043966), cell growth, (GO:0016049, GO:0007033), leaf senescence (GO:0050665), and regulation of intracellular signal transmission (GO:0015693, GO:0016197, GO:1902531). In contrast, genes with higher TF-gene regulatory relationship in the V2-IST network were enriched for biological processes (Supplementary Table 4b) related to cell proliferation and regulation of DNA replication (GO:0000911, GO:0016117, GO:0008283, GO:0006275, GO:0009220, GO:0006281, GO:0042023, GO:0006261), regulation of meristem development (GO:0019953, GO:0048509), chloroplast organization and photosynthesis (GO:0010027, GO:0042793, GO:0009658, GO:0045036, GO:0009657, GO:0016226, GO:0009768, GO:0015979), biosynthesis of amino-acids and regulation of transcription and translation processes (GO:0006520, GO:0006546, GO:0001510, GO:0035304, GO:0016572, GO:0019344, GO:0034660), and other biosynthetic processes, in particular processes related to fatty acids and sugars metabolic processes (GO:0000023, GO:0019252, GO:0006633, GO:0006655). These gene ontology terms reflect the fact that V2-IST is a growing leaf tissue and contains the apical meristem, while husk is a more mature leaf tissue.

To get further insights into the differential regulation of these two tissues, we then sought to identify and biologically characterize tissue-specific TFs-genes regulatory modules. To this end, we obtained two tissue-specific networks, one for husk and one for V2-IST (see section 4 and Supplementary Table 5). We used the tissue-specific TF-gene edges to compute enhancer-gene regulation scores and identify the top gene targets of each enhancer (Supplementary Table 6). This gave us the opportunity to evaluate the proportion of enhancers targeting an immediately flanking gene. We found that it was the case for 52.3% of enhancers active in husk and 58.8% of enhancers active in V2-IST.

**Figure 2:**
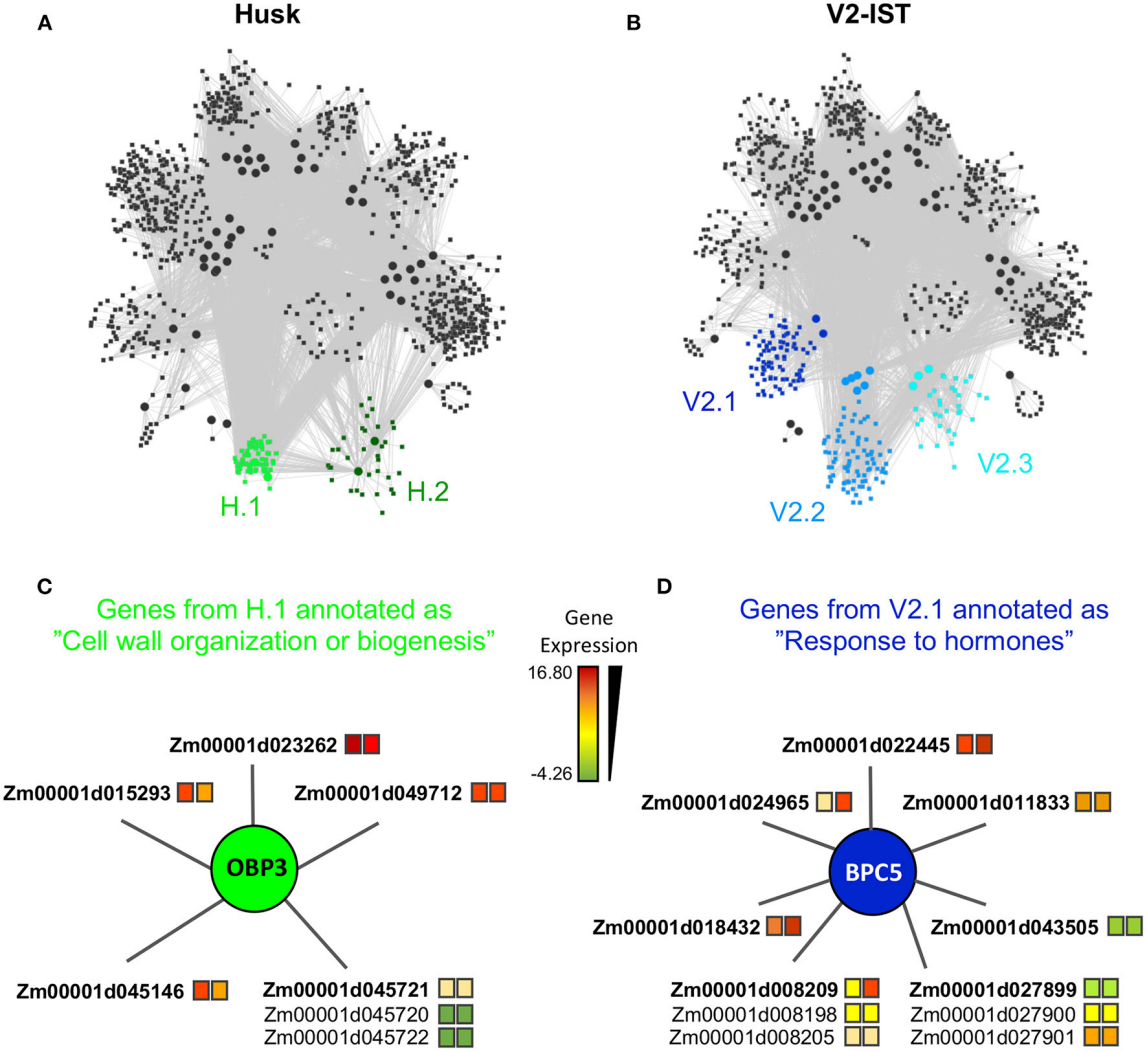
Tissue specific modules. **(A)** Module structure of the husk-specific network. The two husk-specific modules (H.1 and H.2) are highlighted in green. **(B)** Module structure of the V2-IST-specific network. The three V2-IST-specific modules (V2.1, V2.2, and V2.3) are highlighted in blue. **(A,B)** Modules in gray are shared between the husk-specific and the V2-IST specific networks. **(C)** Detailed view of the subset of the husk-specific H.1 module that contains the genes annotated as “cell wall organization or biogenesis”. **(D)** Detailed view of the subset of the V2-IST-specific V2.1 module that contains the genes annotated as “response to hormones”. **(C,D)** Colors of the squares located right to gene IDs indicate average expression levels in husk (left square) and V2-IST (right square). When several genes are potentially targeted by the same enhancer, they are represented with a common edge, and the top target is ranked first and highlighted in bold. The TFs that regulate the genes are represented as circles. Because TFBS annotation arises from *Arabidopsis thaliana*, names of TFs are these of this species. The maize ortholog of *obp3* is *dof27*, that of *bpc5* is *bbr4*. Similar information can be retrieved for all genes of the module using the R application we developed https://maud-fagny.shinyapps.io/TF-gene_network_Maize/.

We then compared the networks structures between Husk and V2-IST using ALPACA (see section 4) (Padi and Quackenbush, 2018). This allowed us to identify regulatory modules (i.e., groups of TFs that were co-regulating groups of genes) in each tissue-specific network and to compare the modules between husk and V2-IST. We identified 71 modules in the V2-IST-specific network, and 67 modules in the husk-specific network. Among them, respectively, 12 and 11 modules contained at least one TF and five genes, and were retained for further investigation. In order to identify shared and tissue-specific modules, we then compared their gene content between husk and V2-IST using the jaccard index. Nine of them had high jaccard indexes (greater than 0.5), indicating that they were very similar between the husk and V2-IST networks (Supplementary Table 7, and gray modules in Figures 2A,B). We then performed Gene Ontology enrichment analysis on genes present in each module (enrichment analysis results are in Supplementary Table 8, and the list of genes in each modules with its Gene Ontology annotation is in Supplementary Table 9). They included 66.8% of the 8,054 genes included in the network prior and contained 570 genes on average (ranging from 5 to 1,269 genes). Gene Ontology enrichment analyses showed that the genes contained in these modules are involved in basic biological functions such as protein metabolism, macromolecular complex organization, and defense response, which are expected to be shared between the two tissues (Supplementary Tables 8a–c).

Besides these nine shared regulatory modules, we found five other modules, which were tissue-specific and included two husk-specific modules (containing 61 and 349 genes, respectively) and three V2-IST-specific modules (containing 319, 811, and 859 genes, respectively, Supplementary Table 7 and Figures 2A,B). These modules tended to be smaller than the shared ones. We next performed a Gene Ontology enrichment analysis on the genes contained in each tissue-specific module, and focused on GO biological processes that were only significant in tissue-specific modules.

**Figure 3:**
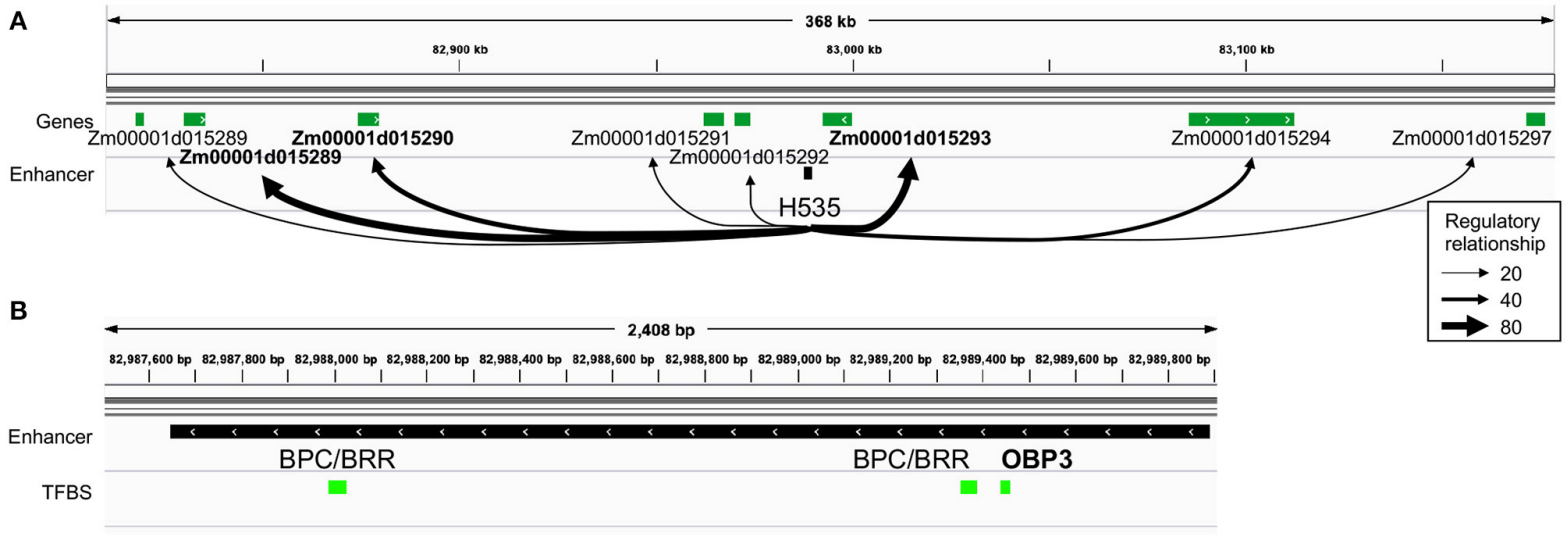
Identification of top targets of a husk-specific enhancer: example of enhancer H535. **(A)** Representation of the regulatory relationships between H535 and all of its potential target genes. Thickness of arrows represents average targeting (regulatory relationship) of the genes by all the TFs potentially binding the enhancer. H535 top target genes are highlighted in bold. **(B)** TFBS content of the H535 enhancer. OBP3 is highlighted in bold, as it is articulating the “heterocyclic compound binding” husk-specific regulatory module that contains Zm00001d015293.

Among the two husk-specific modules, the largest one (349 genes, H.1 in Figure 2A) is enriched for genes involved in “cell wall organization or biogenesis” (GO:0071554, elim algorithm from topGO, *p* = 0.009, Supplementary Table 8d). This module is clustered around OBP3 (ortholog of maize DOF27), a C2C2/DOF TF known to be involved in leaf development and light signalling in maize mature leaves. Its TFBS is enriched within enhancers activated in husk as compared to V2-IST (Supplementary Table 3). It notably regulates *Zm00001d015293* (Figure 2C), a top target of the husk-specific H535 enhancer (Figure 3) known to be involved in leaf development in rice (Zhao et al., 2010) and to be expressed at the basis of mature leaves (Stelpflug et al., 2016; Hoopes et al., 2019). Other interesting targets are *Zm00001d023262* (*brick3*), and *Zm00001d045720/Zm00001d045721/Zm00001d045722*, three genes coding for proteins of the TBL family, which is involved in trichome morphogenesis and secondary cell wall morphogenesis (Figure 2C) (Bischoff et al., 2010). Most of OBP3 target genes are more expressed in husk than V2-IST, as shown in Figure 2C.

The small husk-specific module of 61 genes was particularly interesting (H.2 in Figure 2A). It is enriched in genes involved in the molecular function “heterocyclic compound binding” and “organic cyclic compound binding” (GO:1901363 and GO:0097159, elim algorithm from topGO, *p* = 0.008, Supplementary Table 8e), and it is centered mainly around AT1G12630, a TF from the AP2/EREB family and also includes ERF38, a TF from the DREB subfamily A-4 of the AP2/ERF family (Supplementary Table 6A). Both *erf38* maize ortholog *erf039* and *At1g12630* maize ortholog *ereb10* are over-expressed in husk as compared to V2-IST (log2 fold changes of 2.4 and 0.9, respectively, and Mann–Whitney *U*-test *p*-value of 2.2 × 10^−3^ and 4.1 × 10^−2^, respectively). The expression levels of both of these TFs positively correlate with the expression level of their target genes involved in “heterocyclic compound binding” across all 45 samples and 13 tissues (Supplementary Figures 7, 8). Accordingly, most of these target genes are over-expressed in husk as compared to V2-IST (Supplementary Figure 6A). Finally, ERF38 TFBSs are only found in enhancers that are active in husk but not in V2-IST (Supplementary Table 3), thus highlighting the importance of this TF in husk-specific gene expression regulation.

The largest V2-IST-specific regulatory module (859 genes, V2.1 in Figure 2B) is enriched in genes involved in several biological processes (Supplementary Table 8f) including “sulfur compound biosynthetic process” (GO:0044272, elim algorithm from topGO, *p* = 0.002), “regulation of nucleic acid-templated transcription” (GO:1903506, elim algorithm from topGO, *p* = 0.004) and “response to hormones” (GO:0009725, elim algorithm from topGO, *p* = 3.9 × 10^−2^). In this module, genes classified under the “response to hormones” gene ontology are regulated by BPC5, a member of the BBR/BPC TF family involved in Polycomb complex recruitment (Figure 2D). Consequently, enhancers carrying BPC5 TFBSs are inactive when *bpc5* is expressed and active otherwise. Accordingly, *bpc5* maize ortholog, *bbr4* is less expressed in V2-IST than in husk (log2 fold change −0.4 and Mann–Whitney *U*-test *p* = 2.2 × 10^−3^), its expression is anti-correlated with most of its target genes (Supplementary Figure 9), and its target genes are generally more expressed in V2-IST than in husk (Figure 2D). Notably, its targets include *Zm00001d043505*, a phosphotransmitter (Yonekura-Sakakibara et al., 2004) known to be involved in the response to cytokinin, a hormone promoting cell division. Another target, *Zm00001d008209*, is encoding a protein of the cyclophilin/peptidyl-prolyl cis-trans isomerase family. This gene family is strongly expressed in seedling and growing tissues and is involved in regulating maize development (Marivet et al., 1995).

The second largest V2-IST-specific module (811 genes, V2.2 in Figure 2B) is enriched for genes involved in “cellular macromolecule biosynthetic processes” (GO:0034645, elim algorithm from topGO, *p* = 5.0 × 10^−3^, see Supplementary Figure 6B and Supplementary Table 8g). Finally, the smallest V2-IST-specific module (319 genes, V2.3 in Figure 2B) is enriched for genes involved in “protein complex assembly” (GO:0006461, elim algorithm from topGO, *p* = 7.0 × 10^−3^, see Supplementary Figure 6C and Supplementary Table 8h). Both modules are clustered around TFs of the AP2/ERF family including RAP2-12 for the first one (Supplementary Figure 6B) and ERF5 and ADOF1 for the second one (Supplementary Figure 6C). The corresponding TFBSs are enriched in enhancers activated in V2-IST compared to those activated in husk. Altogether, these results show that the TF-gene regulatory networks that we reconstructed allow to identify tissue-specific regulatory modules whose functions are in agreement with the tissue analyzed, as well as key TFs and their target genes underlying these functions.

### 2.4. Transposable Elements Are a Source of TFBS Sequences in Tissue-Specific Enhancers

**Figure 4:**
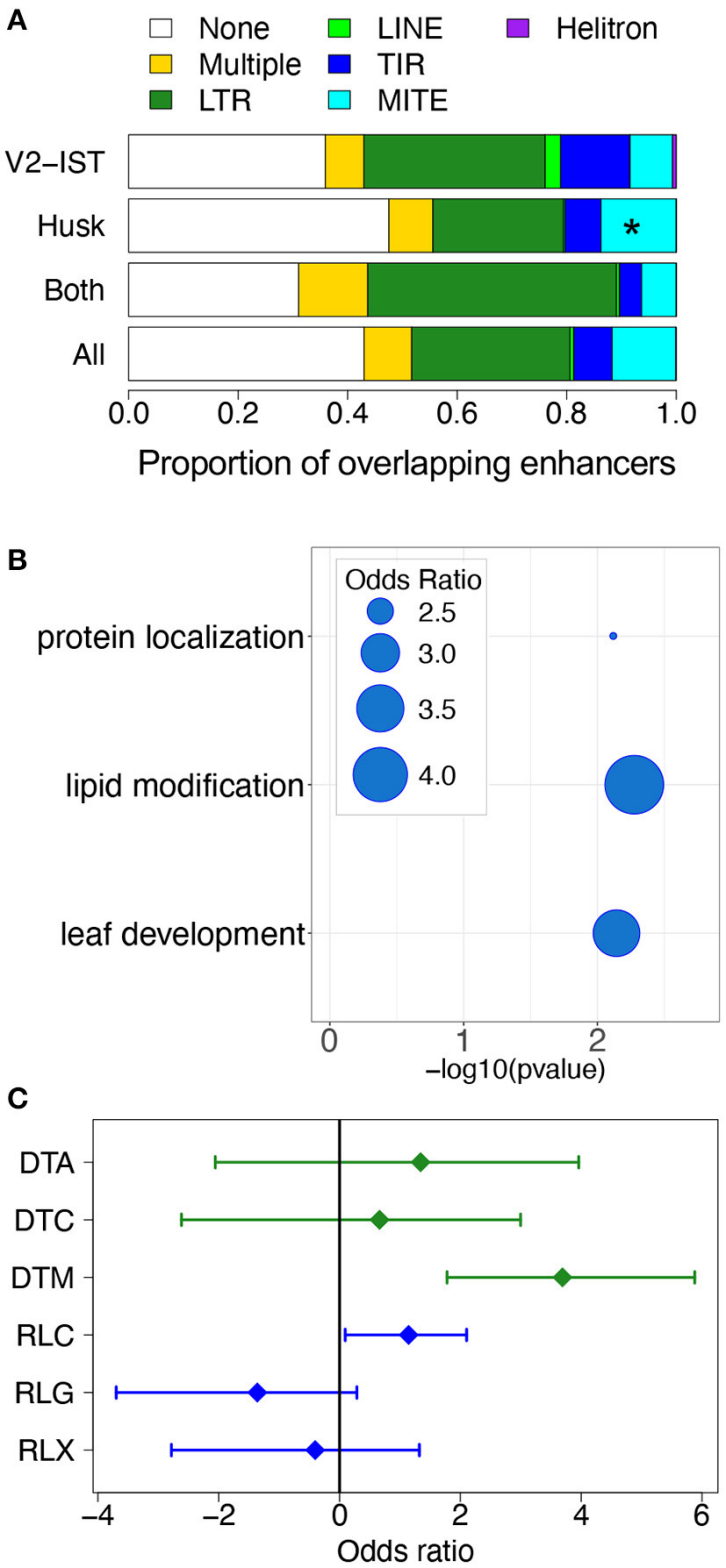
The role of transposable elements in tissue-specific gene expression regulation. **(A)** Proportion of different TE orders in enhancers. “None” corresponds to absence of overlap with a TE, and “Multiple” corresponds to presence of overlaps with TEs from at least two different orders. *p ≤ 0.05. **(B)** Biological functions of genes regulated by husk-specific enhancers overlapping MITEs and carrying potential TFBSs in enhancers. The bubbles represent the odds ratio measuring the enrichment in some Gene Ontology categories among genes potentially targeted by TFs binding the AAGGGATTTYTATTT 15-mer. **(C)** Enrichment in TIR and LTR superfamilies among TFBSs located in V2-IST-specific enhancers. DTA: TIR *hAT*, DTC: TIR *CACTA*, DTM: TIR *Mutator*, RLC: LTR *Copia*, RLG: LTR *Gypsy*, RLX: LTR Unknown.

We took advantage of the regulatory networks and modules we identified to investigate the role of transposable elements (TEs) in the tissue-specific regulation of gene expression. We first compared the TE sequence content of husk-specific and V2-IST-specific enhancers. To this end, we annotated TE in enhancers using a recently updated TE database (Ou et al., 2019). We found that of the 971 enhancers present in the prior, 555 (57.2%) were included in, or partially overlapping at least one TE. On average, when an enhancer overlapped a TE, about 18.7% of the enhancer sequence was covered by the corresponding TE sequence (ranging from 0.4 to 100%). We then tested the relative enrichment in TE superfamilies of husk-specific and V2-IST-specific enhancers (Figure 4A). Because husk-specific enhancers were significantly closer to their nearest genes than V2-IST specific and shared enhancers (average 25,722 and 32,075 bp, respectively,—Mann-Whitney *U*-test one-sided *p* = 7.3 × 10^−3^) and the distribution of TE families is strongly affected by distance to the closest gene, we used randomly chosen genomic sequences on the same chromosome, with the same size and distance to the closest gene than the enhancers to build χ^2^ expected null distributions for each enrichment test (see section 4). We then compared the χ^2^ obtained using the real enhancers with the expected null distribution. We found that husk-specific enhancers are enriched in miniature inverted-repeat transposable elements (MITEs, a group of non-autonomous DNA transposons) as compared to the V2-IST and shared enhancers (odds ratio of 1.8, resampled χ^2^*p* = 0.05).

Most often, however, these MITEs did not overlap with any known TFBSs. Because the JASPAR database mostly contains TFBSs from *A. thaliana*, we hypothesized that this could be due to lack of a relevant maize TFBS motif in the database. Indeed, despite the identification of hundreds of maize TFs (Zhou et al., 2020), JASPAR 2020 only contains 22 maize-specific binding motifs (Khan et al., 2018). To investigate whether the detected MITEs contain a putative TBFS motif, we searched their sequences for conserved motifs by performing an enrichment analysis for 9- to 15-mers (see section 4). We found that MITEs included in husk-specific enhancers are enriched in 3 potential 15 pb TFBS motifs (Supplementary Table 10a): AAATTAGTTYATTTT, AAGGGATTTYTATTT, and GTTCYCAAACTAGCC. By comparing these sequences to known plant TFBS motifs (JASPAR database), we found that they are most closely related to *Arabidopsis* HB-like, MYB-like, and MADS TFBS motifs, respectively (Supplementary Table 10b). These motifs were significantly more present in MITEs of the *Pif/Harbinger* superfamily than in other MITEs superfamilies (odds ratio of 5.4, 9.5, and 9.0, respectively, see Fisher's exact test results in Supplementary Table 11).

To get insights into the potential regulatory role of these enhancers, we investigated the biological functions of the genes targeted by enhancers carrying these motifs (Supplementary Table 12a). Genes targeted by MITE-driven enhancers harboring the AAGGGATTTYTATTT motif are enriched for the biological process “lipid modification” and “leaf development” (see Figure 4B). Genes targeted by MITE-driven enhancers with the AAATTAGTTYATTTT and GTTCYCAAACTAGCC were enriched for “microtubule binding” and “isomerase activity” molecular functions, respectively.

V2-IST-specific enhancers were not enriched in particular TE superfamilies. However, when restricting the analysis to the TFBS parts of enhancers, we found that TFBSs from V2-IST specific enhancers were enriched for TIR transposon *Mutator* (TIR DTM—odds ratio of 12.9, resampled χ^2^*p* = 0.05, Figure 4C). The vast majority (70%) of TFBSs overlapping TIR transposon *Mutator* were from the AP2/ERF family. A gene ontology analysis revealed that candidate targets of enhancers carrying these TFBSs were enriched for biological processes related to nitrogen storage (GO:1901566, GO:0009073, and GO:0044283, see Supplementary Table 12b).

## 3. Discussion

In this study, we investigated the regulatory relationship between TFs and target genes in two leaf tissues at different developmental stages: V2-IST at seedling stage, and husk (bracts) at flowering. To do so, we used a bipartite network-based approach integrating several layers of information from heterogeneous data: epigenetic information (providing genomic location of active enhancers), genomic information (providing gene-candidate enhancer distance and annotation of TFBSs within enhancers) and transcriptomic information from 13 different tissues sampled at different developmental stages. This allowed us to provide functional insights into the regulatory role of enhancers that are activated in a tissue-specific manner.

Several approaches are routinely used to analyze gene regulatory networks. Classic weighted co-expression networks allow for the detection of genes that are co-expressed in the same tissues as well as modules corresponding to major biological functions. However, they do not detect which regulators are responsible for this co-expression (as has been shown in Sonawane et al., 2019). In addition, co-expression networks can generate erroneous results due to the presence of spurious correlations between genes that are not involved in the same biological functions (Sonawane et al., 2017; Parsana et al., 2019). TF-gene regulatory interactions can also be inferred using bipartite TF-gene networks that are based on correlations between the expression of TFs and their target genes across tissues. Such networks have proven powerful to study regulatory networks for different maize tissues and to identify key regulatory TFs across development (Zhan et al., 2015; Walley et al., 2016; Huang et al., 2018) or in response to the environment (Kimotho et al., 2019; Zhou et al., 2020). However, they have the same drawback as the gene co-expression networks—because they do not include information about regulatory sequences, they are also susceptible to spurious correlations. In addition, TFs whose expression patterns are not correlated with their targets will not be captured (Sonawane et al., 2019). The approach we use integrates information about enhancer sequences and gene co-expression in a tissue-specific manner. In these networks, high edges between a TF and its genes indicate not only that the genes are co-expressed, but also that there are enhancers near these genes that carry the same TFBS. This method thus allows the identification of groups of co-regulated genes and corresponding key regulatory TFs. Here, these are the specific interactions that are involved in the regulation of leaf-specific functions at two different developmental stages. In addition, our methodology also allowed to identify the putative enhancers that regulate the expression of target genes. This has two major advantages: (i) by characterizing the enhancers involved, it allows to investigate the molecular origin of tissue-specific regulation, and (ii) by identifying candidate enhancer-target genes pairs, it reduces the number of candidate target genes for each enhancer, thus limiting the number of candidates to be tested using molecular biology approaches. Moreover, while our results show that most enhancers target an immediately flanking gene, we also predict that about 42–48% of enhancers skip at least one gene, thus highlighting the complexity of the maize genome organization and gene expression *cis*-regulation.

Our study allowed us to identify regulatory modules corresponding to functions relevant to the tissue analyzed. For instance, in V2-IST, we find molecular functions expected to be found in an immature and growing tissue such as “cellular macromolecule biosynthetic processes,” “protein complex assembly” and “hormone response.” While we consolidate existing results, we also provide the key players (i.e., groups of co-regulated genes and their key regulatory TFs) involved in these functions. In addition to the to-be-expected modules, our approach also allowed us to discover regulatory modules of more unclear function, notably in husk. Husks are the bracts of the maize female inflorescence that provide a mechanical protection of the ear and growing silks (the styles of maize florets). In particular, husks ensure silks growth by protecting them from air evaporative demand and preserving their water status (Fuad-Hassan et al., 2008). Its other biological functions, if any, remain poorly characterized. While the largest module, containing genes involved in the biosynthesis of cell walls, has been previously described in leaves in a late developmental stage (Li et al., 2010), we identified a yet to be characterized husk-specific module. This module is regulated by two TFs, ERF039 and EREB10, whose functions are unknown in maize. Nevertheless, based on studies in other species, they are likely to be involved in response to biotic and abiotic stresses (Kimotho et al., 2019; Xie et al., 2019). For instance, an ortholog of ERF039 (also ortholog to *A. thaliana* ERF38) is involved in salt and osmotic tolerance in poplar (Cheng et al., 2019). Moreover, the AP2/EREB family, to which EREB10 belongs, participates in response to abiotic stress in soybean and maize (Marcolino-Gomes et al., 2013; Waters et al., 2017). Their candidate targets include genes coding for TFs involved in the modification of photosynthesis and photomorphogenesis in response to abiotic stresses in many plant species (Kazan, 2013; Liu et al., 2015; Wang et al., 2016; Han et al., 2020). Husk growth is a component of the anthesis to silking interval, which is a good predictor of grain yield under stress (Bolaños and Edmeades, 1996). Silk emergence out of the husk to be pollinated is indeed known to result from the balance between silk and husk growth rates, which are both responsive to abiotic constraints. Drought tolerance in maize thus partly relies on the coupling of tissue expansion in both vegetative and reproductive organs (Turc et al., 2016). Our findings further support the importance for husk growth to be regulated in response to abiotic constraints, and pinpoint the genes and TFs involved in this process, thus allowing for their further molecular characterization. This will be particularly useful to understand how to improve maize response to drought.

In our attempt to characterize biological functions expressed in a tissue with limited biological characterization, we nevertheless encountered two limitations. First, the lack of functional annotation of maize genes in public databases prevented us to precisely annotate some of the husk-specific regulatory modules. Second, our TFBSs prediction in maize enhancers is based on motifs included in the JASPAR Plantae motifs database, which are mainly derived from ChIP-seq experiments performed in *Arabidopsis thaliana*. We validated our TFBS prediction for one TF using published DAP-seq data (the only one for which data were available), thus suggesting that the JASPAR database is relevant to detect TFBS in maize genome. Nevertheless, we are still likely to be missing a number of tissue-specific functions regulated by maize-specific TFs. While some progress toward the identification of maize TFBS motifs has been made through the use of sequence conservation analyses (Tian et al., 2019), this limitation can only be circumvented by addition of new maize ChIP-seq- or DAP-seq-derived motifs to the TFBS databases, which is likely to occur in the near future. Despite these two caveats, we were able to provide candidate TFs and genes playing a key role in the expression of husk-specific biological functions. Our approach, coupled with the rapidly increasing data available on maize-specific TFBS motifs (Burgess et al., 2019), will thus improve our capacity to concomitantly identify enhancers, TFs and genes involved in the regulation of the expression of biological functions in poorly characterized tissues.

Our results support the role of TEs as functional actors in the tissue-specific regulation of biological functions involved in leaf differentiation. We find that a substantial amount (~60%) of the enhancers analyzed include TE sequences. This is higher than estimated in previous studies (Oka et al., 2017; Zhao et al., 2018) and likely arises from the fact that we used an upgraded maize TE database (Ou et al., 2019), which allowed a more in depth characterization of TEs within enhancers. Several studies have shown that TEs can modify gene regulation under stress conditions, for instance in rice (Naito et al., 2009) and in maize (Makarevitch et al., 2015). But the underlying mechanisms are still unclear. Several cases of TE-driven enhancers involved in the regulation of specific genes have been described in plants. For instance, a *Hopscotch* LTR retrotransposon regulates the domestication gene *tb1* in maize through a long-range interaction (Studer et al., 2011; Ricci et al., 2019). In *A. thaliana*, LINE *EPCOT3* is involved in the neo-functionalization of the *Cyp82c2* gene, thus contributing to chemical diversity and pathogen defense (Barco et al., 2019). *In Brassica napus*, a CACTA transposon acts as an enhancer to stimulate expression of the *BnaA9.CYP78A9* gene and silique elongation (Shi et al., 2019). In maize, a recent analysis of chromatin accessibility at the genome-wide level revealed that TEs, in particular LTR retrotransposons, contribute to gene regulation as *cis*-regulatory elements (Zhao et al., 2018). But this study did not connect TE-driven enhancers to their target genes.

Here, by taking advantage of the concomitant characterization of enhancers and their target genes, we discovered the TE sequences associated with the enhancers, but also the functions that they regulate. This allowed us to show that TIR transposons and MITEs can directly regulate gene expression through their domestication as enhancers in maize, and are involved in the regulation of tissue-specificity. Interestingly, while Zhao et al. (2018) pointed mainly to the role of LTR retrotransposons, we point here to the role of TIR transposons and MITEs in tissue-specific regulation, suggesting that these elements may be involved in tissue-specificity. Moreover, we show that two distinct TE families, TIR transposon *Mutator* and MITE *Pif/Harbinger*, have provided TFBSs to enhancers regulating the expression of genes from two distinct pathways: nitrogen storage in V2-IST, and late-stage leaf development in husk, respectively. This highlights potential selection of different families to rewire regulatory networks across development.

Finally, through analysis of MITE *Pif/Harbinger*-driven enhancers, we discovered a new potential TFBS motif involved in the regulation of husk development, which is likely recognized by a MYB-like TF. MYB-like TFs have been shown to be highly expressed in the late stage of maize leaf development, and to play a role in the regulation of circadian rhythm and photosynthesis regulation (Yu et al., 2015). Here, we propose that part of the gene regulatory network underlying late-stage husk development has been shaped by the domestication of MITE elements carrying MYB-like TFBSs. Our results complete previous ones showing that the transposition of MITEs have helped amplify specific TFBS and rewire the gene regulatory networks controlling key biological processes in several species, including the response to stress and flowering time in peach and other *Prunus*, and fruit ripening in tomato (Morata et al., 2018). This highlights the power of our methodology to identify new potential TFBSs.

To conclude, our combined analysis of maize enhancer functional annotation, gene-enhancer genomic distance and transcriptomic data using bipartite networks allowed us to analyze the role of enhancers in the development of leaf tissues. We were able to identify key actors involved in leaf development at different molecular levels, from the biological functions involved, to the underlying enhancer-target gene pairs and key transcription factors. We highlighted the role of TIR transposable elements as important actors of tissue-specific gene regulatory expression wiring, through their domestication as distal *cis*-regulatory sequences. We also discovered new potential TE-based TFBSs. By connecting enhancers to their target genes, and identifying the biological functions they potentially regulate, our work opens new avenues to study the impact of enhancer structural variation on the wiring of gene regulatory networks and, ultimately, to the underlying phenotype.

## 4. Materials and Methods

### 4.1. Previously Generated Data

We use the coordinates of active enhancers and corresponding mRNA-seq data from two different tissues, husk (the soft inner leaves surrounding the ear) and V2-IST (the inner part of leaves 3 and onwards of stage V2 seedlings) from the B73 maize line. Complete description of these tissues is presented in Supplementary Table 1. These data were generated in a former work (Oka et al., 2017). Briefly, active enhancer coordinates were obtained by intersecting DNAse I hypersensitivity DNAse-seq, histone mark H3K9ac ChIP-seq and DNA methylation bisulfite-sequencing profiles (see Oka et al., 2017). RNA-seq data for six replicates for both husk and V2-IST were also provided by Oka and colleagues (raw fastq files with 100 bp single-end reads). More information about plant growth, RNA extraction and library preparation can be found in Oka et al. (2017).

### 4.2. Generation of mRNA-Seq Data: mRNA Extraction, Library Preparation, and Sequencing

We generated 150 bases paired-end mRNA-seq data from 11 tissues. Tissue types, growing conditions and sampling are summarized in Supplementary Table 1. For all tissues, mRNAs were extracted from three independent plants (biological triplicate), except for hypocotyl and roots, where a replicate is a pool of three different plants and for 17DAP and 35DAP seeds, where a replicate is a pool of seeds from a single ear. For leaf, internode, silk, tassel, immature ear and 17DAP seed, RNAs were isolated with Trizol (Invitrogen ref.15596018) and β-mercaptoethanol (SIGMA ref. M3148-25ML) reagents. Supernatant was recovered and RNA purified using Qiagen RNeasy Plant Mini kit (ref. 74904) following manufacturer's instructions. Then, a Qiagen RNAse-free DNAse set (ref. 79254) was applied to remove the residual DNA. A different protocol was used for 35DAP seed mRNA extraction: RNAs were extracted with 4.5 ml of buffer (10 mM Tris-HCl, pH 7.4, 1 mM EDTA, 0.1 M NaCI, 1% Sodium Dodecyl Sulfate) and 3 ml of phenol—chloroform—isoamyl alcohol mixture 25:24:1. The supernatant was extracted one more time with the same phenol solution in order to eliminate proteins and starch. The nucleic acids were precipitated by addition of 0.1 vol of 3M sodium acetate pH 5.2 and 2 vol of 100% ethanol. After precipitation RNA were rinsed one time with 70% ethanol and the pellets dissolved in RNase-free water. Purification was done with a DNAse treatment RNase-Free DNase Set (Qiagen, Hilden, Allemagne) and then RNeasy MinElute Cleanup Kit (Qiagen, Hilden, Allemagne). Quality of total RNA samples was assessed using the Agilent 2100 bioanalyzer (California, USA) according to manufacturer's recommendations. Library construction was generated by the IPS2-POPS platform. Briefly, mRNAs were polyA selected, fragmented to 260 bases and libraries were built using the TruSeq stranded mRNA kit (Illumina®, California, U.S.A.) with an Applied BioSystem 2720 Thermal Cycler and barcoded adaptors. Barcoded libraries were sequenced on Illumina HiSeq4000 at Genoscope, in paired-end (PE) with 150 bases read length. Approximately 20 millions of paired-end reads were produced for each sample.

### 4.3. RNA-Seq Data Pre-processing and Normalization

Raw illumina reads (fastq) were trimmed with Trimmomatic (Bolger et al., 2014) tool for Phred Quality Score Qscore >20, read length >30 bases, and ribosome sequences were removed with tool sortMeRNA (Kopylova et al., 2012). They were then aligned to the AGPv4 version of the B73 maize genome using STAR (Dobin et al., 2012) and the following options:—runMode alignReads—alignIntronMin 5—alignIntronMax 60000—runThreadN 32—readFilesCommand gunzip -c—quantMode GeneCounts SortedByCoordinate—outSAMprimaryFlag AllBestScore—outFilterMultimapScoreRange 0—outFilterMultimapNmax 20—alignEndsType Local—sjdbGTFtagExonParentGene gene_id. Read counts per gene were calculated with STAR from reads unambiguously mapped on genes. With these settings, over all tissues, 91.1% of reads (median) were mapped unambiguously to a gene for the Oka et al. dataset (ranging from 87.9 to 92.1%), and 95.8% for the AMAIZING Gene Atlas dataset (ranging from 92.8 to 97.7%).

Counts per gene from both datasets were then pooled and normalized together using the tissue-aware smooth quantile normalization from the R bioconductor *YARN* package version 1.1.1 (Paulson et al., 2017), using the normalizeTissueAware function with the *method*= “*qsmooth*” option. Data were then corrected for single-end/paired-end batch effect using the removeBatchEffect function from the R bioconductor *limma* package version 3.28.2.

### 4.4. Enhancer Definition and TFBS Identification

Candidate enhancer sequences were extracted from the bed files containing coordinates of enhancers from Oka et al. (2017) and the AGPv4 maize genome sequence using bedtools getfasta. They were scanned for Transcription Factor Binding Sites (TFBS) using the *FIMO* software from the *MEME* v. 5.0.5 suite (Grant et al., 2011) using default parameters. To this end, we retrieved the MEME core plants position frequency matrix files corresponding to the binding sites of 489 transcription factors available in JASPAR database (accession October 31, 2019) (Khan et al., 2018). Matches with a *q*-value (Benjamini–Hochberg corrected *p*-value) lower than 0.01 were retained.

Significance of the TFBS results was tested by comparing the number of bases covered by TFBSs in original candidate enhancer sequences to this of random sequences obtained by shuffling enhancers dinucleotides. To this end, for each enhancer, we generated 1,000 random sequences using the BiasAway software (Worsley Hunt et al., 2014) and computed resampling *p*-values by counting the number of random sequences for which TFBS coverage exceeded the one of the original enhancer.

We compared the TFBS motif content of husk and V2-IST enhancers by using the AME software from the *MEME* suite version 5.0.5 (McLeay and Bailey, 2010). Enrichment in particular TFBSs among husk enhancers was estimated by setting husk as primary and V2-IST as background sequences. This procedure was swapped to obtain V2-IST enhancer TFBS enrichment. We tested enrichment for motifs using the Fisher exact test, and *p*-values were corrected for multiple testing using the Bonferroni method. *E*-value threshold was set to default (*E* ≤ 10).

### 4.5. TF-Gene Network Building

We built tissue-specific regulatory networks using the PANDA and LIONESS softwares (Glass et al., 2013; Kuijjer et al., 2019). PANDA represents regulatory relationships between TFs and genes as a bipartite network, nodes being either TFs or genes, and edge weight being proportional to the strength of the TF-gene regulatory relationship. The method requires as input a prior representing potential regulatory relationships between TFs and genes. The prior is a gene × TFs matrix of zeros and ones, were ones indicate the presence of a putative TFBS in a *cis*-regulatory region of the gene, and zero its absence. This prior edge matrix is then updated using a protein-protein interaction (PPI) matrix that represents interactions between TFs and a gene co-expression matrix. This relies on a message-passing algorithm that verifies both the “responsibility” and the “availability” of each edge (Glass et al., 2013). The final PANDA output is an “aggregate” network model representing gene regulation in a specific dataset. LIONESS is a mathematical framework that extracts networks for individual samples from such an aggregate regulatory network (Kuijjer et al., 2019).

In this study, the prior TF-gene interaction matrix was obtained by crossing enhancer coordinates with gene coordinates. The gene-enhancer maximal distance was set up to 250kb. This threshold is based on analysis of the enhancer-nearest gene distance distributions of Ricci et al. (2019) and Lu et al. (2019), which show that 99% of distances are below 250 kb. This allows us to capture a large fraction of distal cis-regulatory elements, while keeping the number of genes and enhancers to analyze at a computationally acceptable amount. All enhancers within 250 kb upstream or downstream of a gene transcription start site were annotated as a potential regulator, and prior edges between this gene and each transcription factor mapping to those enhancers were set to 1. All other prior edges were set to 0. The co-expression matrix was obtained from the mRNA-seq normalized data from the 45 samples (13 tissues). In absence of a detailed protein-protein interaction matrix for plants, we used an identity matrix. Using PANDA and LIONESS, we generated 46 networks: a global network (PANDA), and 45 sample-specific ones (LIONESS). Raw sample-specific edges weights (*EW*) were log-transformed (*logEW*) using the following formula: for each sample *i* and edge *e*:

$$\begin{array}{l} logEW_{ei}=\ln\left(\;exp\left(\;EW_{ei}\;\right)+1\;\right) \end{array}$$

Edges that were set to 0 in the prior were set to 0 in the all the following analyses.

Enhancer-gene TSS distances thus obtained were also used to compute the distributions of distances between enhancers and nearest gene TSS. Briefly, the closest gene was the one whose TSS was the closest from the gene in absolute value.

### 4.6. Identification of Most Likely Target Gene

In order to identify the most likely target gene of each enhancer, we computed the average edge weight for each enhancer-potential target gene pair. For each pair of enhancer *e* and potential target gene *g*, the average edge weight (*avgEW*_*g*_) was computed as the average of the edges (logEW) from each TF *t* to the gene. Only TFs with a potential factor binding site in the enhancer were included. With *T*_*e*_ the number of TFBSs in the enhancer:

$$avgEW_{g}=\frac{\sum_{t}logEW_{tg}}{T_{e}}$$

### 4.7. Tissue-Specific Gene Targeting

To identify genes that were differentially targeted between husk and V2-IST tissues, edges weights were compared between tissues-conditions using a linear regression performed with the R bioconductor *limma* R package version 3.28.2.

$$EW_{gr}\sim \underset{k}{\sum }\beta_{kg}\times TissueCondition_{kr}+\epsilon_{gr}$$

*k* is the tissue-condition and *r* the replicate.

Genes that were targeted by edges with Benjamini–Hochberg corrected *p*-values under 0.01 were considered as differentially targeted.

### 4.8. Identification of Tissue-Specific TF-Genes Regulatory Modules

To identify tissue-specific regulatory modules, we first build one husk-specific and one V2-IST-specific network by averaging the LIONESS sample-specific networks across the replicates for each tissue. We then identified the TF and genes that changed the modularity of the networks between V2-IST and husk by running ALPACA (Padi and Quackenbush, 2018), setting in turn the V2-IST and husk networks as background. This function outputs one list of regulatory modules—i.e., groups of TFs that regulate groups of genes based on the edge weights—for each tissue-specific network. We then compared the gene content of the regulatory modules between husk and V2-IST by computing pairwise jaccard indexes. The maximum jaccard index was conserved for each module in each tissue. Modules with maximum jaccard index over 0.5 were annotated as shared between tissues, and modules with maximum jaccard index under 0.5 were annotated as tissue-specific.

### 4.9. Gene Ontology Enrichment Analyses

We performed Gene Ontology enrichment analyses using the R bioconductor topGO package (Alexa and Rahnenfuhrer, 2016), using the elim method. In this approach, that has been proved more efficient than the classic Fisher test (Alexa et al., 2006), the tests are not independent, as the GO categories are tested one after the other, following the GO tree structure from bottom to the top. When one category in one level of the GO tree is significant, the genes involved are removed (eliminated) form the subsequent tests. Because the tests are not independent, no multiple testing correction can be applied. Instead, following the guidelines from the users' manual, we filtered uncorrected *p*-values using a stringent threshold of 0.01. We also filtered out all categories that did not include at least 5 genes in the gene set of interest. For GO enrichment analysis of genes in tissue-specific modules, if the module contained more than 100 genes, we performed gene enrichment analyses on the 100 genes that were the most connected to TFs within the regulatory module (top differential modularity genes from the ALPACA results). The gene ontology database used in this analysis was generated by combining publicly available annotations (Wimalanathan et al., 2018) obtained from InterproScan5, Arabidopsis and uniprot from https://datacommons.cyverse.org/browse/iplant/home/shared/commons_repo/curated/Carolyn_Lawrence-Dill_maize-GAMER_maize.B73_RefGen_v4_Zm00001d.2_Oct_2017.r1/d.non_red_gaf (January 2020), and removing any redundancy.

### 4.10. Annotation of Transposable Elements in Enhancers and TFBS

We annotated the transposable elements of the B73 genome (AGPv4) using REPEATMASKER v4.0 (http://www.repeatmasker.org) with an updated TE database provided by Shujun Ou (https://github.com/oushujun/EDTA/blob/master/database/maizeTE11122019, Ou et al., 2019). We annotated enhancers and TFBSs using this database and the data.table R package (Dowle and Srinivasan, 2016).

We tested for enrichment in a particular transposable element superfamily among husk-specific or V2-IST-specific enhancers using a χ^2^-test. In order to take into account the genomic location of the enhancers in the TE enrichment analysis, we computed a null distribution of χ^2^ values using 1,000 resamplings of genomic sequences with same length, chromosome and distance to nearest gene TSS as the original list of enhancers used.

### 4.11. Motif Discovery in MITE Sequences

We searched MITE sequences overlapping husk-specific enhancers for motifs using the MEME software (Bailey and Elkan, 1994). Because TFBSs from plants are typically 11 nt long, we searched for motifs of length 9–15 nt. We filtered out motifs with an *E*-value over 10^−4^. We then used the online version of Tomtom (Gupta et al., 2007) to compare these motifs with known TFBS motifs available in the JASPAR 2018 non-redundant core plants database. We filtered out all motifs with a *p*-value greater than 0.01.

## Data Availability Statement

All steps of the experiment, from growth conditions to bioinformatic analyses, were managed in CATdb database (Gagnot et al., 2007) (http://tools.ips2.u-psud.fr/CATdb/) with Project ID NGS2017 06 AMAIZING, experiment Amaizing B73 WW. This project has been submitted from CATdb into the international repository GEO (Gene Expression Omnibus, Edgar et al., 2002; http://www.ncbi.nlm.nih.gov/geo) with ProjetID GSE151455. These data are available upon request to Clementine Vitte, and will be made publicly available upon article acceptance.

## Author Contributions

CV, MF, and MS conceived the experiments. CV and JJ secured the funding. CV and MF performed the experiments and analyzed the data. OT coordinated greenhouse culture, sampling strategy, and sample collection. AV, CV, JJ, and OT collected the samples. AV performed the tissue grinding and mRNA extractions. SP generated the mRNA-seq libraries. JR aligned the mRNA-seq reads. MF and CV wrote the article. JJ, MK, MS, and OT provided the expertise and feedback and corrected the manuscript. All authors contributed to the article and approved the submitted version.

## Conflict of Interest

The authors declare that the research was conducted in the absence of any commercial or financial relationships that could be construed as a potential conflict of interest.
