# Supplementary material for: Identification of Key Tissue-Specific, Biological Processes by Integrating Enhancer Information in Maize Gene Regulatory Networks
Source: Front Genet. 2021 Jan 11;11:606285. doi: 10.3389/fgene.2020.606285 (PMC7834273; doi:10.3389/fgene.2020.606285)
Supplement: Supplementary file 13 [file Data_Sheet_1.pdf]

Supplementary Material

SUPPLEMENTARY DATASETS AND FIGURES

Supplementary Datasets

Table S1. Supplementary_Dataset_S1_Nomenclature_samples.xlsx. Samples characteristics.

Table S2. Supplementary_Dataset_S2_enhancers_annotation_TFBS.xlsx. Annotation of Husk and V2-IST enhancers with JASPAR 2019 core Plants database.

Table S3. Supplementary_Dataset_S3_TFBS_enrichment_enhancers.xlsx. Binding sites mapping preferentially in husk or V2-IST enhancers.

Table S4. Supplementary_Dataset_S4_difftarget_GO_enrichment.xlsx. Gene Ontology enrichment analysis of genes preferentially targeted in (a) HUSK and (b) V2-IST.

Table S5. Supplementary_Dataset_S5_tissue-specific_networks.xlsx. Tissue-specific networks corresponding to HUSK and V2-IST. These networks are queryable on our app `https://maud-fagny.shinyapps.io/TF-gene_network_Maize/`.

Table S6. Supplementary_Dataset_S6_toptarget_enhancers.xlsx. List of enhancers and top target genes.

Table S7. Supplementary_Dataset_S7_jaccard_index_communities.xlsx. Comparison of the gene content of husk and V2-IST regulatory modules using jaccard index.

Table S8. Supplementary_Dataset_S8_modules_GO_enrichment.xlsx. Gene Ontology enrichment analysis of modules in HUSK-specific and V2-IST specific networks. It contains 8 subtables that correspond to Gene Ontology Terms enrichment among genes of (a-c) the three shared modules, (d-e) the two HUSK-specific modules H.1 and H.2, and (f-h) the three V2-IST-specific modules V2.1, V2.2, V2.3.

Table S9. Supplementary_Dataset_S9_genes_GO_annotation_by_module.xlsx. List of genes present in each module of HUSK and V2-IST with their Gene ontology annotation.

Table S10. Supplementary_Dataset_S10_Husk_MITE_TFBS.xlsx. Prediction of new TFBS motifs in MITEs elements overlapping husk-specific enhancers. (a) Predicted TFBS motifs in each MITE sequence. (b) Comparison of the predicted TFBS motifs with known TFBS motifs.

Table S11. Supplementary_Dataset_S11_MITEs_superfamilies_motifs_enrichment.xlsx. Enrichment analysis of MITE putative TFBS motifs among *Pif/Harbinger* elements.

Table S12. Supplementary_Dataset_S12_enhancers_TE_target_GO.xlsx. Gene Ontology enrichment analysis of genes targeted by enhancers containing TE. (a) GO enrichment for genes targeted by husk-specific enhancers overlapping MITEs and carrying each of the 3 motifs for HUSK. (b) GO enrichment for genes targeted by husk-specific enhancers overlapping TIR Mutator for V2-IST.

Supplementary Figures

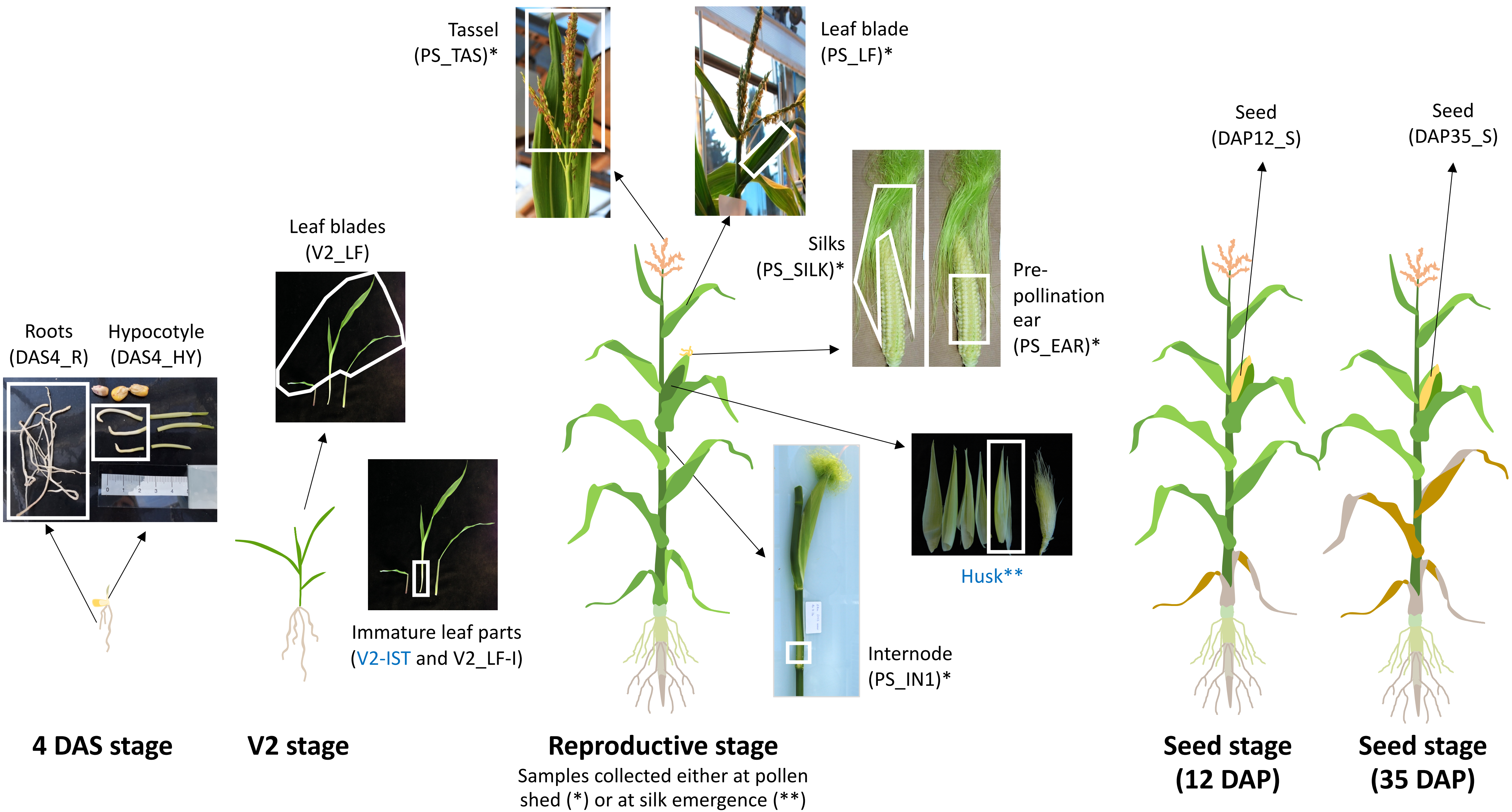
Tassel
(PS_TAS)*
Leaf blade
(PS_LF)*
Seed
(DAP12_S)
Seed
(DAP35_S)
Leaf blades
(V2_LF)
Silks
(PS_SILK)*
Pre-
pollination
ear
(PS_EAR)*
Roots
(DAS4_R)
Hypocotyle
(DAS4_HY)
Husk**
Immature leaf parts
(V2-IST and V2_LF-I)
Internode
(PS_IN1)*
4 DAS stage
V2 stage
Reproductive stage
Samples collected either at pollen shed (*) or at silk emergence (**)
Seed stage
(12 DAP)
Seed stage
(35 DAP)

Figure S1. Description of the tissues sampled. Plant drawings show the global features of a maize plant for each of the 5 developmental stages analyzed. Black arrows point to the tissues sampled, and, for each tissue, pictures show the portion of the tissue that was sampled (area surrounded by a white frame). Sample names are shown in brackets. At reproductive stage, all samples were collected at pollen shed (one asterisk), except husk that was collected at silk emergence (two asterisks). Before silk emergence, ears were bagged to avoid pollination, thus allowing to collect pre-pollination ears and silks. Prior to ear dissection, silks were cut just above husk tip, in order to restrict sampling to the growing part of silk tissues enclosed by husks. The two tissues corresponding to the datasets from Oka *et al.*., 2017 (*i.e.*, V2-IST and husk) are highlighted in blue. Detailed description of the samples and corresponding datasets is available in Supplementary Table S1. DAS: Days after sowing. DAP: Days After Pollination.

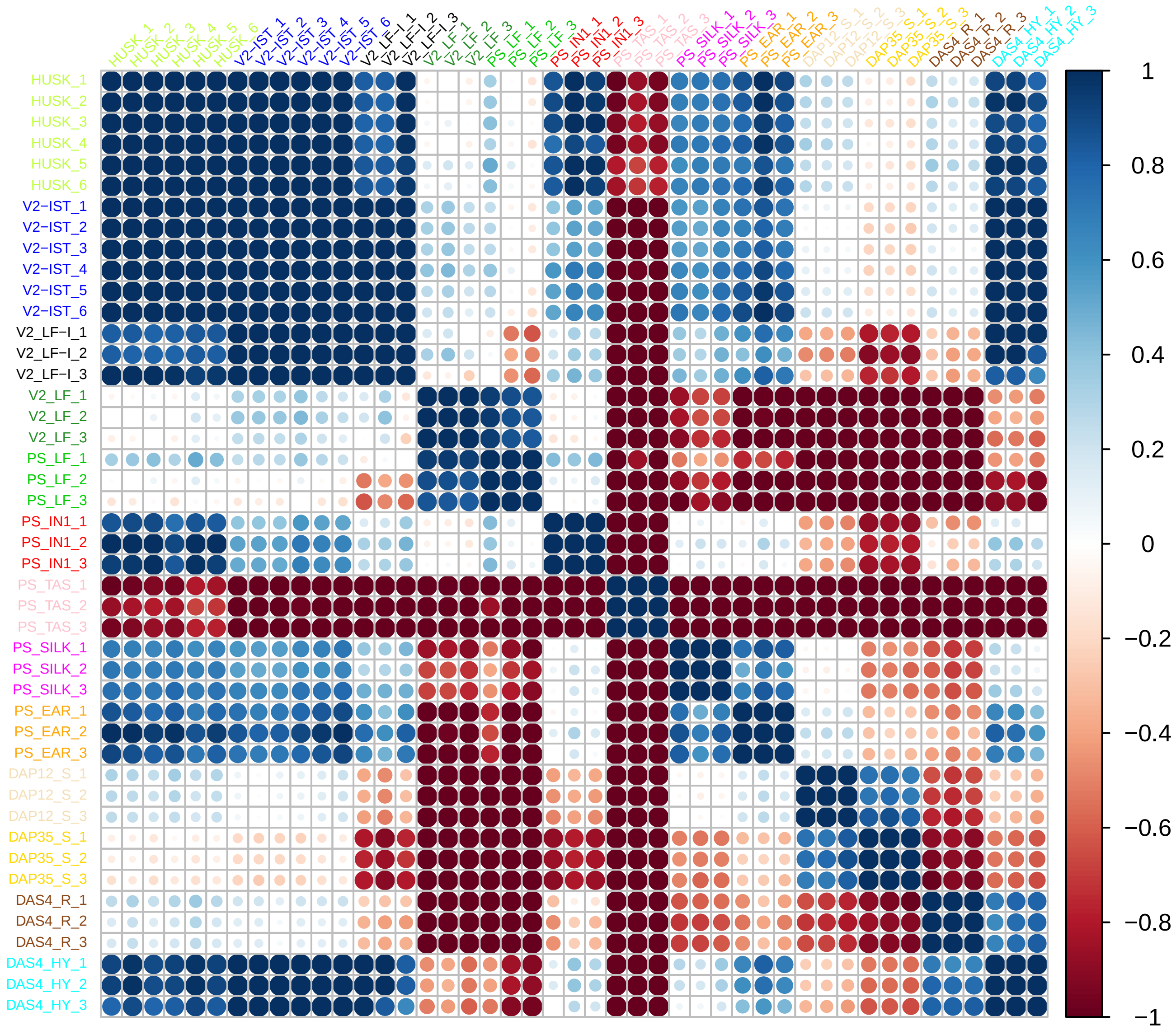

Figure S2. Gene Expression Correlation. The matrix represent the Pearson's R obtained by performing pairwise expression correlation between all samples on filtered and normalized data.

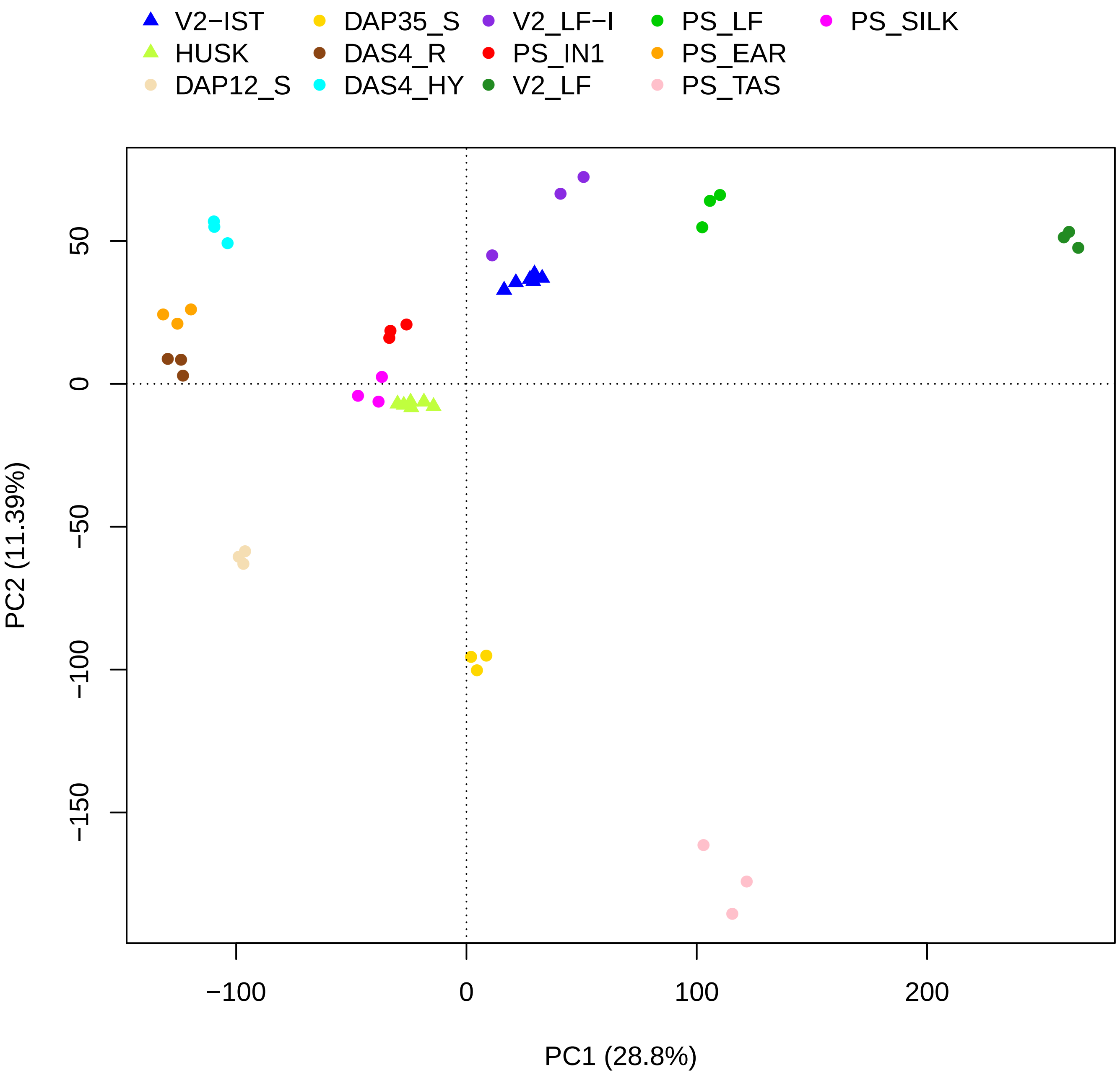

Figure S3. Principal component analysis of normalized and batch-corrected RNA-seq expression data from the GeneAtlas AMAIZING dataset (circles) and Oka *et al.* dataset (triangles).

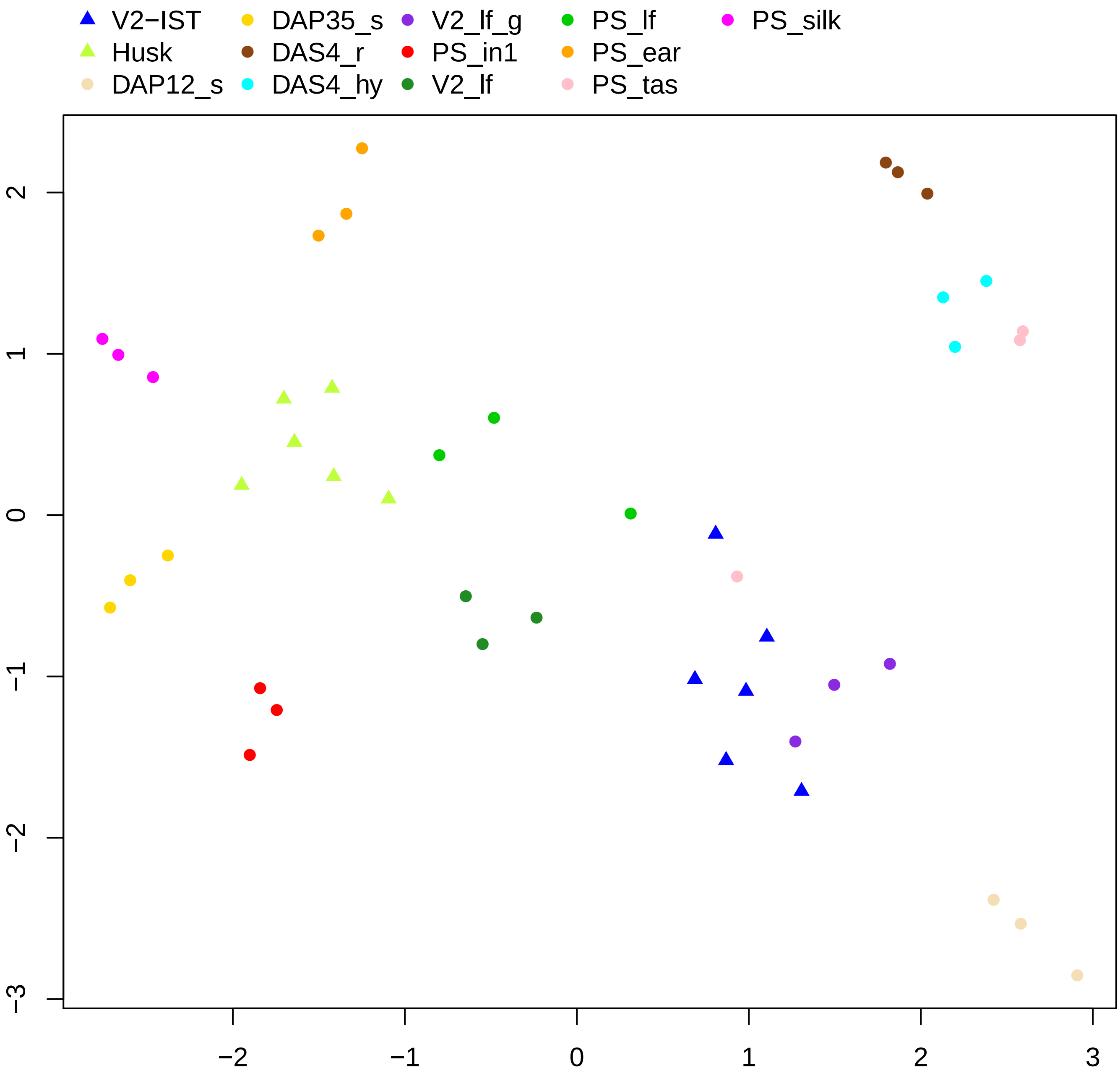

Figure S4. UMAP representation of edge weights from sample-specific networks. Circles correspond to samples from the GeneAtlas AMAIZING dataset and triangles to the Oka *et al.* dataset.

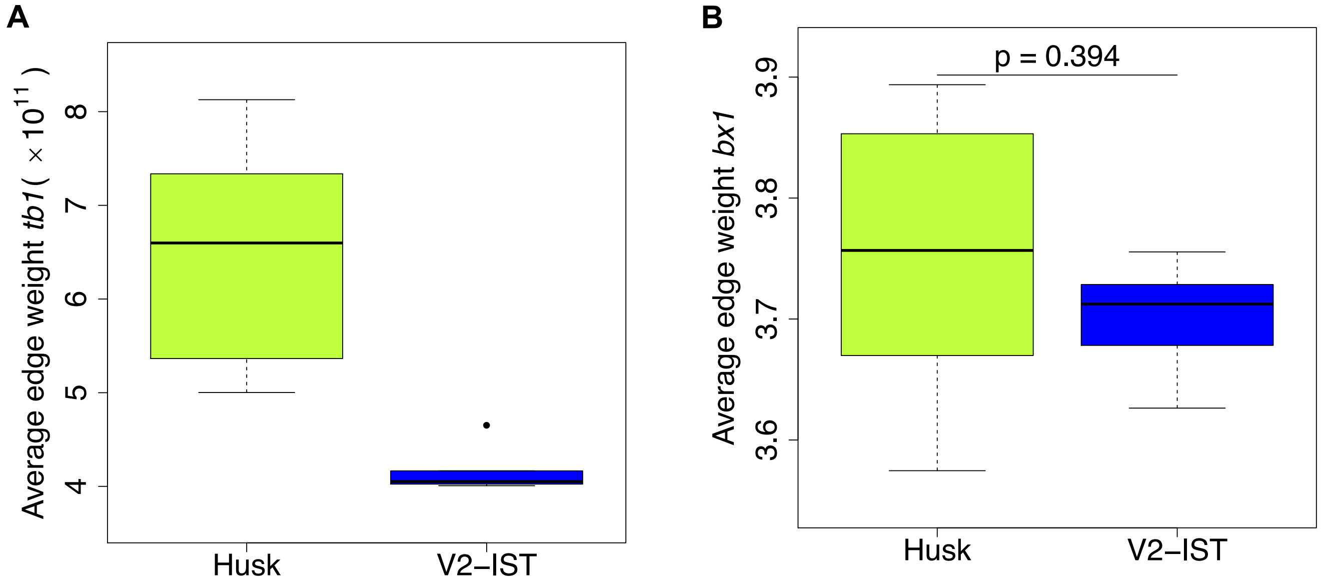

Figure S5. Average edge weights between known enhancers and their target genes. **A.** Enhancer of *tb1*, which is active in husk but not in V2-IST: distribution of average edge weight between *tb1* and the transcription factors that have binding sites in its enhancer for husk and V2-IST samples. **B.** Enhancer of *bx1* (DICE), which is found to be active in both husk and V2-IST: distribution of the average edge weight between *bx1* and the transcription factors that have binding sites in DICE for husk and V2-IST samples.

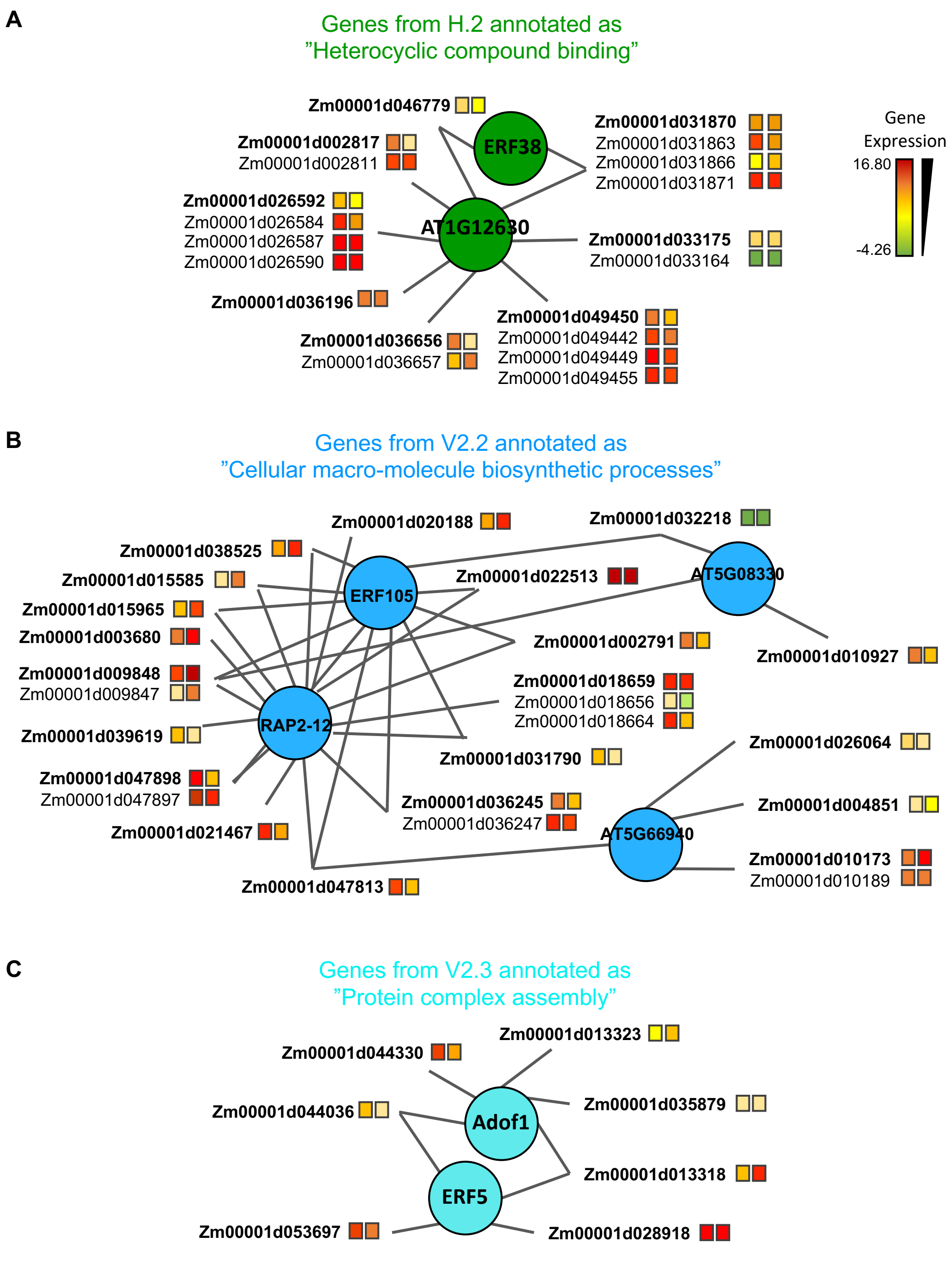

Figure S6. Detailed view of the husk and V2-IST-specific modules. textbfA. Detailed view of the part of the husk-specific H.2 module that shows the genes annotated as "heterocyclic compound binding". **B.** Detailed view of the part of the V2-IST-specific V2.2 module that shows the genes annotated as "cellular macromolecule biosynthetic processes", showing the connection of all the genes involved in this biological process. **C.** Detailed view of the part of the V2.3 module that shows the genes annotated as "protein complex assembly", showing the connection of all the genes involved in this biological process. **A-C.** The color of the squares beside the names represent the genes average expression levels in husk (left square) and V2-IST (right square). When several genes are potentially targeted by the same enhancer, they are represented with a common edge, and the top target is ranked first and highlighted in bold. The TFs that regulate the genes are represented as circles. Because TFBS annotation arise from *Arabidopsis thaliana*, names of TFs are these of this species. Maize orthologs of *erf38*, *At1g12630*, *rap2-12 erf5* and *adof1* are *erf039*, *ereb10*, *bbr4*, *ereb210*, *ereb61*, and *dof7* respectively. Similar information can be retrieved for all genes of the module using the R application we developped `https://maud-fagny.shinyapps.io/TF-gene_network_Maize/`.

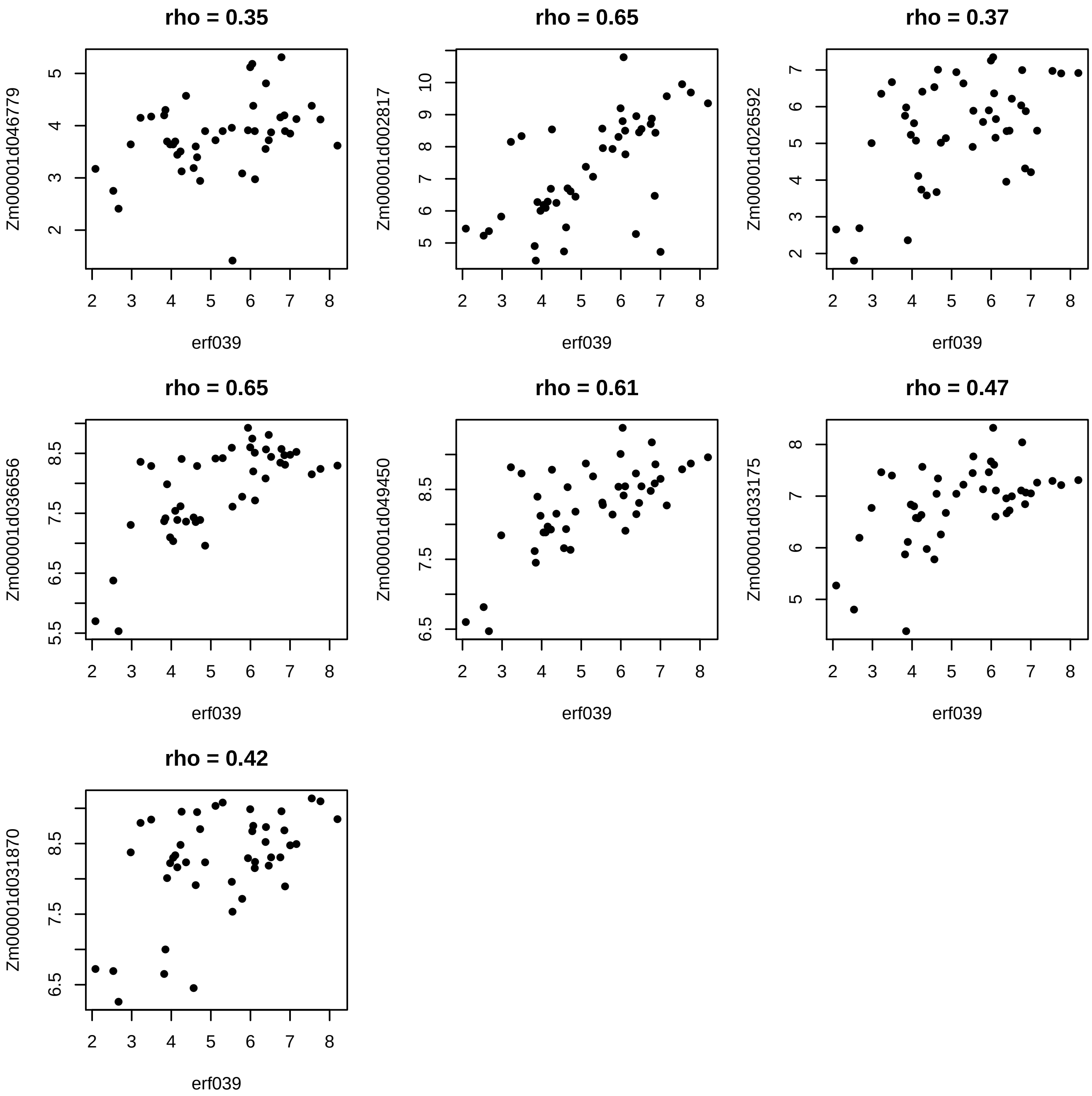

Figure S7. Correlations of expression levels between *erf039*, the maize ortholog of *erf38* and ERF38 target genes involved in heterocyclic compound binding across all 45 samples. Only the top target gene for each ERF38-binding enhancer is represented. Each panel represents the correlation between *erf039* (x-axis) and one of its target gene annotated as ”heterocyclic compound binding” (y-axis). Spearman’s rho values are indicated on top of each graph.

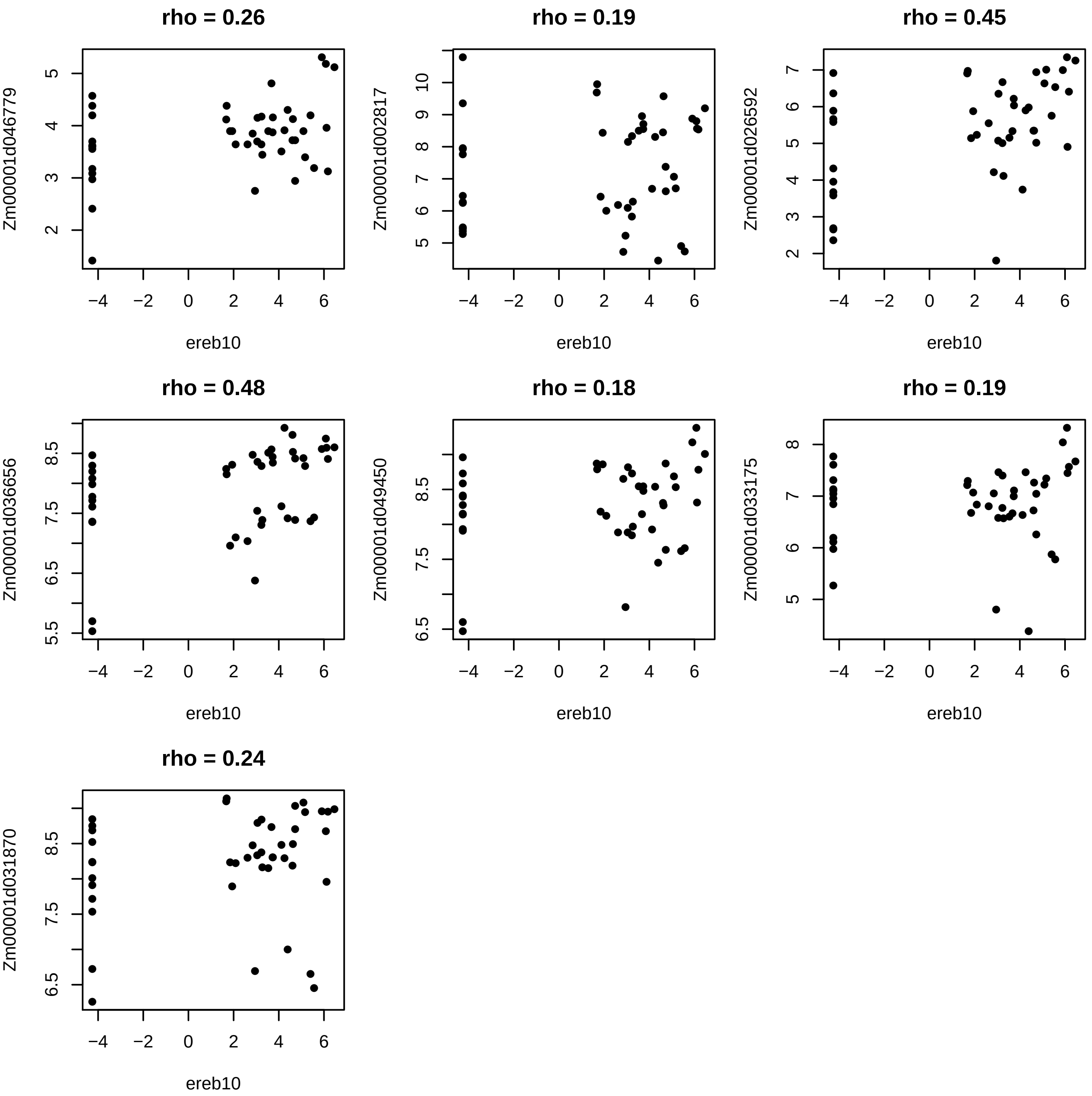

Figure S8. Correlations of expression levels between *ereb10*, the maize ortholog of *At1g12630* and AT1G12630 target genes involved in heterocyclic compound binding across all 45 samples. Only the top target gene for each AT1G12630-binding enhancer is represented. Each panel represents the correlation between *ereb10* (x-axis) and one of its target genes annotated as ”heterocyclic compound binding” (y-axis). Spearman’s rho values are indicated on top of each graph.

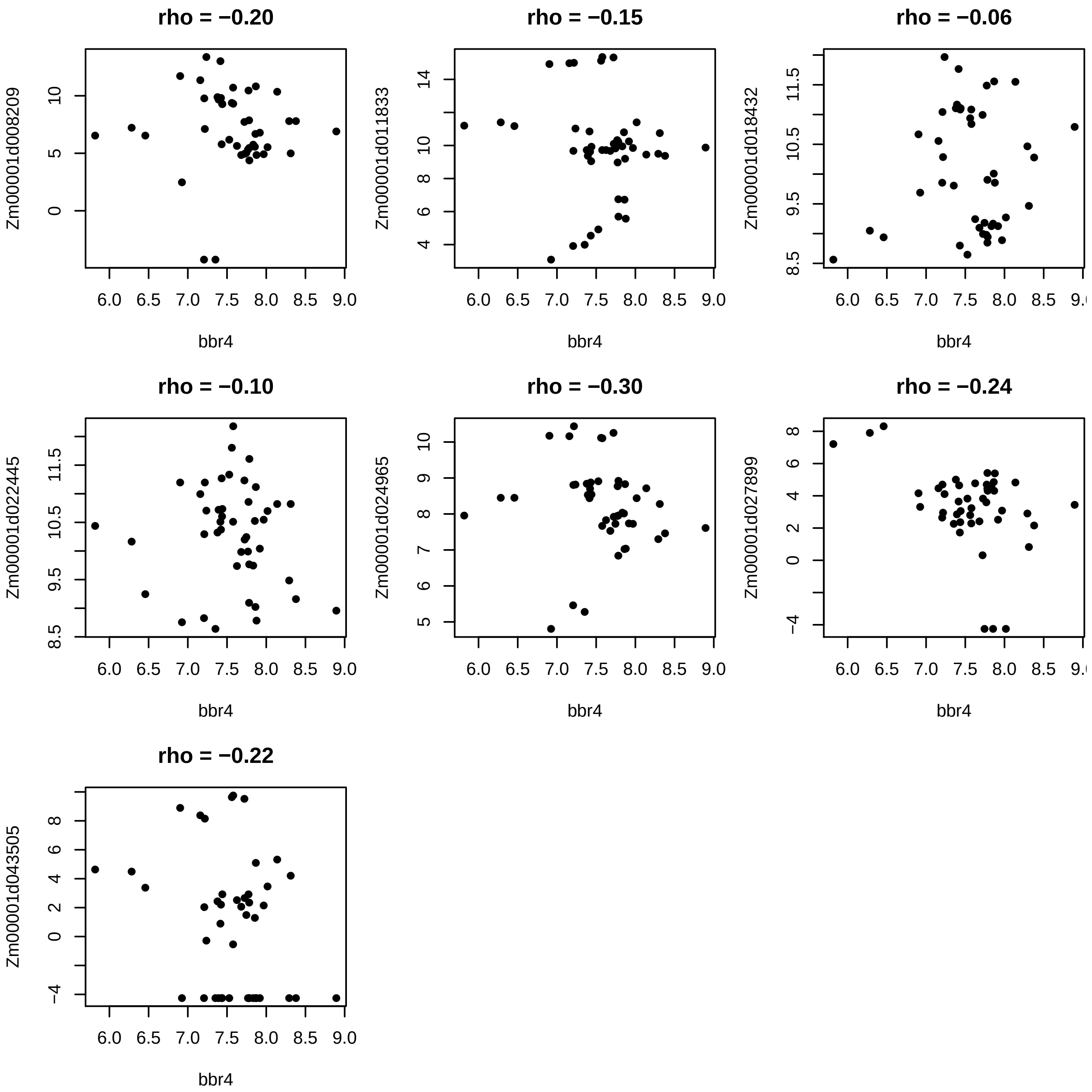

Figure S9. Correlations of expression levels between *bbr4*, the maize ortholog of *bpc5*, and BPC5 target genes involved in hormone response across all 45 samples. Only the top target gene for each BPC5-binding enhancer is represented. Each panel represents the correlation between *bbr4* (x-axis) and one of its target genes annotated as ”hormone response” (y-axis). Spearman’s rho values are indicated on top of each graph.
